# Insufficiency of phosphatidylethanolamine *N*-methyltransferase is risk for lean non-alcoholic steatohepatitis

**DOI:** 10.1038/srep21721

**Published:** 2016-02-17

**Authors:** Atsuko Nakatsuka, Makoto Matsuyama, Satoshi Yamaguchi, Akihiro Katayama, Jun Eguchi, Kazutoshi Murakami, Sanae Teshigawara, Daisuke Ogawa, Nozomu Wada, Tetsuya Yasunaka, Fusao Ikeda, Akinobu Takaki, Eijiro Watanabe, Jun Wada

**Affiliations:** 1 Department of Medicine and Clinical Science, Okayama University Graduate School of Medicine, Dentistry and Pharmaceutical Sciences, Kita-ku, Okayama 700-8558, Japan; 2 Shigei Medical Research Institute, Minami-ku, Okayama 701-0202, Japan; 3 Department of General Medicine, Okayama University Graduate School of Medicine, Dentistry and Pharmaceutical Sciences, Kita-ku, Okayama 700-8558, Japan; 4 Department of Diabetic Nephropathy, Okayama University Graduate School of Medicine, Dentistry and Pharmaceutical Sciences, Kita-ku, Okayama 700-8558, Japan; 5 Department of Gastroenterology and Hepatology, Okayama University Graduate School of Medicine, Dentistry and Pharmaceutical Sciences, Kita-ku, Okayama 700-8558, Japan; 6 Dainippon Sumitomo Pharma, 2-6-8 Doshomachi, Chuo-Ku, Osaka 541-0045, Japan

## Abstract

Although obesity is undoubtedly major risk for non-alcoholic steatohepatitis (NASH), the presence of lean NASH patients with normal body mass index has been recognized. Here, we report that the insufficiency of phosphatidylethanolamine *N*-methyltransferase (PEMT) is a risk for the lean NASH. The Pemt−/− mice fed high fat-high sucrose (HFHS) diet were protected from diet-induced obesity and diabetes, while they demonstrated prominent steatohepatitis and developed multiple liver tumors. Pemt exerted inhibitory effects on p53-driven transcription by forming the complex with clathrin heavy chain and p53, and Pemt−/− mice fed HFHS diet demonstrated prominent apoptosis of hepatocytes. Furthermore, hypermethylation and suppressed mRNA expression of F-box protein 31 and hepatocyte nuclear factor 4α resulted in the prominent activation of cyclin D1. PEMT mRNA expression in liver tissues of NASH patients was significantly lower than those with simple steatosis and we postulated the distinct clinical entity of lean NASH with insufficiency of PEMT activities.

Non-alcoholic fatty liver disease (NAFLD) has been widely recognized as an important manifestation of metabolic syndrome and the development of non-alcoholic steatohepatitis (NASH) is closely related to the obesity and insulin resistance^1^. Although obesity is undoubtedly one of the main risk factors for the development of NAFLD, many clinical observations demonstrated the presence of lean NAFLD patients with normal body mass index (BMI) and the prevalence of lean NAFLD was 12% in Greece^2^, 20% in India^3^ and 15% in China^4^ in recent reports. The lean NAFLD is now recognized as a major cause of cryptogenic liver disease^5^. It is also believed that the accumulation of visceral adipose tissue is main source of fatty acids, proinflammatory and profibrogenic mediators and it closely related to the progression of NASH^6^,^7^. Unexpectedly, recent reports have suggested the accumulation of visceral adipose tissues is not a major determinant for the steatosis, inflammation and fibrosis of the liver in the patients with NAFLD^8^,^9^,^10^. These studies raised the important issues regarding the presence of distinctive clinical entity of lean NAFLD and NASH, although widely acceptable etiology-based classification is not available so far^11^. There are some animal models and genetic diseases, which lead to the hepatic steatosis without obesity and insulin resistance in rodents and human. For instance, the methionine and choline-deficient diet (MCD) mouse model has been widely used as a standard research tool for the investigation of NASH, however, the animals fed MCD do not develop obesity and insulin resistance, and they rather lose weight. Previous studies demonstrated that liver fat accumulation is a consequence of reduce β-oxidation and decreased release of very low density lipoproteins (VLDL)^12^. Recently, it has been reported that the reduction of white adipose tissues (WAT) is associated with increased phosphorylation of hormone-sensitive lipase, and up-regulation of genes encoding carboxylesterase 3 and β2-adrenergic receptor in WAT, where lipolysis is enhanced in MCD mice^13^. Methionine deficiency (MD) treatment decreased glucose and increased fibroblast growth factor 21 (FGF21) in serum, thus exhibiting a similar metabolic phenotype as the fasting response^13^,^14^. Transgenic mice overexpressing acyl-CoA:diacylglycerol acyltransferase 2 (DGAT2), which catalyzes the final step of triacylglycerol (TG) biosynthesis, is another instance and they developed hepatic steatosis with increased amounts of TG, diacylglycerol, ceramides, and unsaturated long-chain fatty acyl-CoAs in the liver, but they had no abnormalities in insulin sensitivity and glucose metabolism^15^. In human, an allele in PNPLA3 (rs738409[G] encoding patatin-like phospholipase-3/adiponutrin I148M) was strongly associated with increased hepatic fat levels^16^, inflammation and fibrosis in the patients with NAFLD^17^. However, recent report demonstrated that the G allele in PNPLA3 rs738409 increases the risk of NAFLD in the general population, especially in subjects without metabolic syndrome, independent of dietary pattern and metabolic factors^18^. Similarly, hepatic steatosis is not only induced by genetic predisposition but also by malnutrition, kwashiorkor, and malabsorption such as pancreaticoduodenectomy^19^, celiac disease^20^, and inflammatory bowel disease^21^.

Phosphatidylethanolamine *N*-methyltransferase (PEMT) is an enzyme that catalyzes the methylation of phosphatidylethanolamine (PE) to phosphatidylcholine (PC) as well as conversion of S-adenosylmethionine (SAM) to S-adenosylhomocystein (SAH)^22^ and PC is synthesized from choline via CDP-choline pathway and liver cells can also synthesize PC via Pemt pathway^23^. Here, we demonstrated that the insufficiency of Pemt is a risk for the lean NAFLD. The Pemt−/− mice fed high fat-high sucrose (HFHS) chow were protected from diet-induced obesity and diabetes, while they demonstrated prominent steatohepatitis and developed multiple liver tumors in current study. The hepatocytes in Pemt−/− mice fed HFHS chow revealed prominent apoptosis and proliferation activities. We further demonstrated that the formation of tri-complex of Pemt, clathrin heavy chain (CHC) and p53 have inhibitory effects on p53-driven transcription and the deficiency of Pemt resulted in enhanced transcriptional activities of p53-driven genes in Pemt−/− mice fed HFHS chow. Furthermore, Pemt−/− mice demonstrated the enhanced methylation of genomic DNA and reduction of mRNA expressions of F-box protein 31 (Fbxo31) and hepatocyte nuclear factor 4α (HNF4α) revealed by methylation analysis of liver genomic DNA with next generation sequencing and DNA microarray analysis. Finally, PEMT mRNA expression in liver tissues of NASH patients was significantly lower than simple steatosis and we postulated presence of distinct clinical entity of lean NASH with insufficiency of PEMT activities.

## Result

### Pemt deficiency resists to diet-induced obesity and insulin resistance

At 25 weeks of age, the diet-induced bogy weight gain observed in Pemt+/+ and Pemt+/− mice fed HFHS chow was completely reversed in Pemt−/− mice to the levels of the mice fed STD chow (Supplementary Fig. 1a). Epididymal fat weight of Pemt−/− mice fed HFHS chow was also reduced to the level of the mice fed STD chow (Supplementary Fig. 1b). Glucose tolerance test and insulin sensitivity test demonstrated that insulin resistance was significantly ameliorated in Pemt−/− mice compared to Pemt+/+ mice under HFHS chow (Supplementary Fig. 1d–i). *Ad libitum* and fasting blood glucose, total cholesterol and triglyceride levels in sera were lower in Pemt−/− mice compared to Pemt+/+ mice under HFHS chow (Supplementary Fig. 2a–d). Serum adiponectin levels were not altered in mice fed HFHS chow by the deficiency of Pemt gene (Supplementary Fig. 2e). Serum leptin levels significantly increased in Pemt+/+ and Pemt+/− mice fed HFHS chow compared with those fed STD chow. In Pemt−/− mice fed HFHS chow, the leptin levels were down-regulated to basal levels observed in the mice fed STD chow (Supplementary Fig. 2f).

### Pemt−/− mice fed HFHS chow develop steatohepatitis and multiple liver tumors

**Figure 1:**
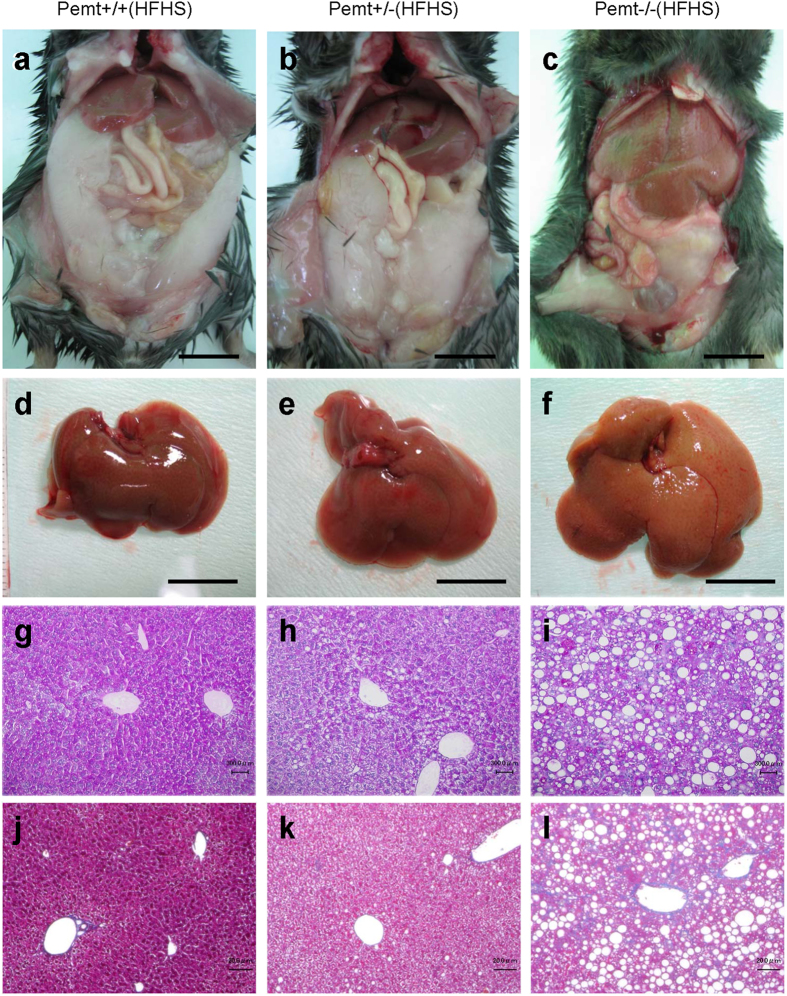
Phenotype of Pemt+/+, Pemt+/− and Pemt−/− mice under high fat-high sucrose (HFHS) diet at 25 weeks of age. (**a**–**c**) Gross appearance of liver and epididymal fat pad. Bar = 1 cm. (**d**–**f**) Gross appearance of dissected liver tissues. Bar = 1 cm (**g**–**i**). Periodic acid-Schiff staining of liver tissues. Bar = 300 μm (**j**–**l**). Masson-Trichrome staining of liver tissues. Bar = 200 μm. Liver tissues in Pemt−/− mice fed HFHS diet demonstrated prominent accumulation of large lipid droplets, infiltration of mononuclear cells and fibrosis.

**Figure 2:**
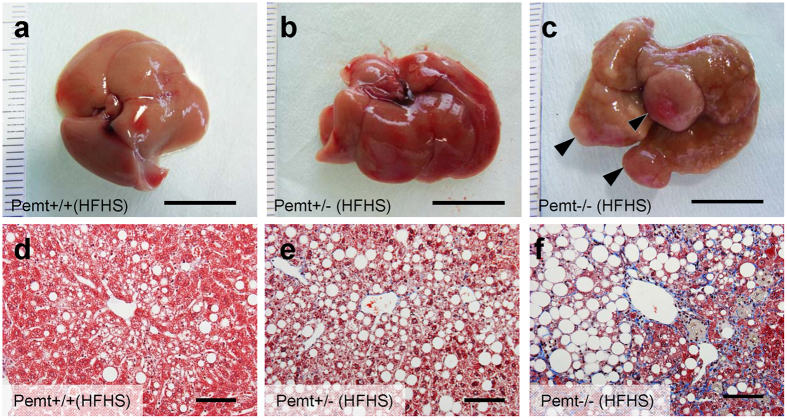
Phenotype of Pemt+/+, Pemt+/− and Pemt−/− mice under high fat-high sucrose (HFHS) diet at 60 weeks of age. (**a**–**c**) Gross appearance of liver. Bar = 1 cm. Regenerative nodules and adenoma are indicated by arrow heads (**c**). (**d**–**f**) Masson-Trichrome staining of liver tissues. Bar = 100 μm.

**Figure 3:**
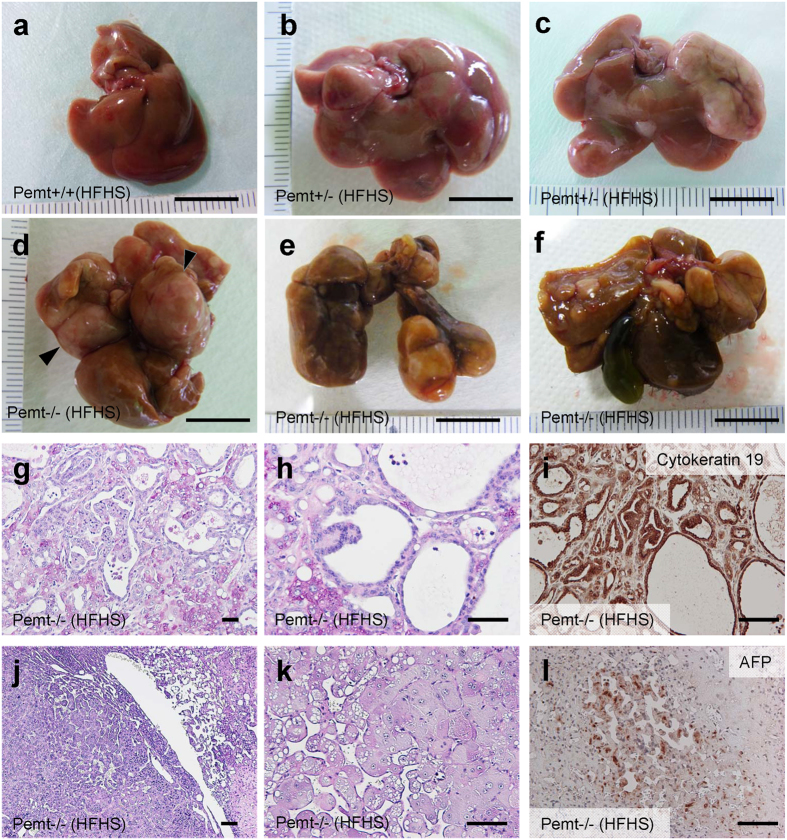
Phenotype of Pemt+/+, Pemt+/− and Pemt−/− mice under high fat-high sucrose (HFHS) diet at 90 weeks of age. (**a**–**f**) Gross appearance of liver in Pemt+/+ (**a**), Pemt+/− (**b**,**c**), Pemt−/− (**d**–**f**) mice. Bar = 1 cm. (**g**–**i**) Cholangiocellular carcinoma in the liver of Pemt−/− mice. Periodic acid-Schiff staining (**g**,**h**) and immunoperoxidase staining with cytokeratin 19 antibodies (**i**). Bar = 100 μm. (**j**–**l**) Regenerative liver tissue in Pemt−/− mice. Periodic acid-Schiff staining (**j**,**k**) and immunostaining with anti-α fetoprotein (AFP) (**l**). Bar = 100 μm.

Pemt−/− mice fed HFHS chow demonstrated prominent hepatomegaly and they were characterized as yellowish appearance, uneven surface and firm consistency (Supplementary Fig. 1c, Fig. 1a–f). In light microscopy, the accumulation of large lipid droplets and infiltration of mononuclear cells were observed in the liver of Pemt−/− mice fed HFHS chow (Fig. 1g–i). F4/80 positive macrophages significantly increased in Pemt−/− mice fed HFHS chow (Supplementary Fig. 3a) and hepatic fibrosis was enhanced revealed by Masson-Trichrome staining (Fig. 1j–l). The expressions of α-smooth muscle actin (αSMA) and transforming growth factor β (TGFβ) were also accentuated in the liver of Pemt−/− mice fed HFHS (Supplementary Fig. 3b). By electron microscopic observation of hepatocytes, glycogen granules were readily observed in Pemt+/+ fed HFHS chow. In contrast, increased number of large lipid droplets and drastic reduction of glycogen granules were observed in Pemt−/− mice fed HFHS chow. (Supplementary Fig. 4a,b). Oval-shaped mitochondria associated with loosely arranged cristae increased in Pemt−/− mice fed HFHS chow compared with Pemt+/+ mice fed STD chow. Further increased number and volume of mitochondria were demonstrated in hepatocytes of Pemt−/− mice fed HFHS chow (Supplementary Fig. 4c,d). Lamellar structure of rough ER was exclusively observed in Pemt+/+ mice fed HFHS chow, while round-shaped smooth ER was dominant in Pemt−/− mice fed HFHS chow. In the extracellular space, interstitial collagen fibers were seen in Pemt−/− mice fed HFHS chow. These ultrastructural alterations in hepatocytes were similar to methionine- and choline-deficient diet and streptozotocin-induced diabetic rodent models in previous reports^24^,^25^. We further investigated the liver histopathology for the extended periods of 60- to 90-week of age. Notably, all of Pemt−/− mice under HFHS chow developed multiple liver tumors at 60-week of age (Fig. 2a–c). Histological investigations revealed that they were regenerative nodules and adenomas. In the background of non-tumor tissues, the characteristic features of progressive steatohepatitis were observed such as accumulation of larger lipid droplets, infiltration of inflammatory cells and prominent fibrosis (Fig. 2d–f). At 90-week of age, multiple tumors progressively grew larger and became dumbbell-shape in Pemt−/− mice, while no tumors were observed in Pemt+/+ mice fed HFHS chow (Fig. 3a–f). AFP positive regenerative nodules were frequently seen in Pemt−/− mice fed HFHS (Fig. 3g–i) and cytokeratin 19 positive cholangiocellular carcinoma developed in ~10% of Pemt−/− mice fed HFHS (Fig. 3j–l).

### Enhanced apoptosis and proliferation of hepatocytes in Pemt−/− mice fed HFHS chow

**Figure 4:**
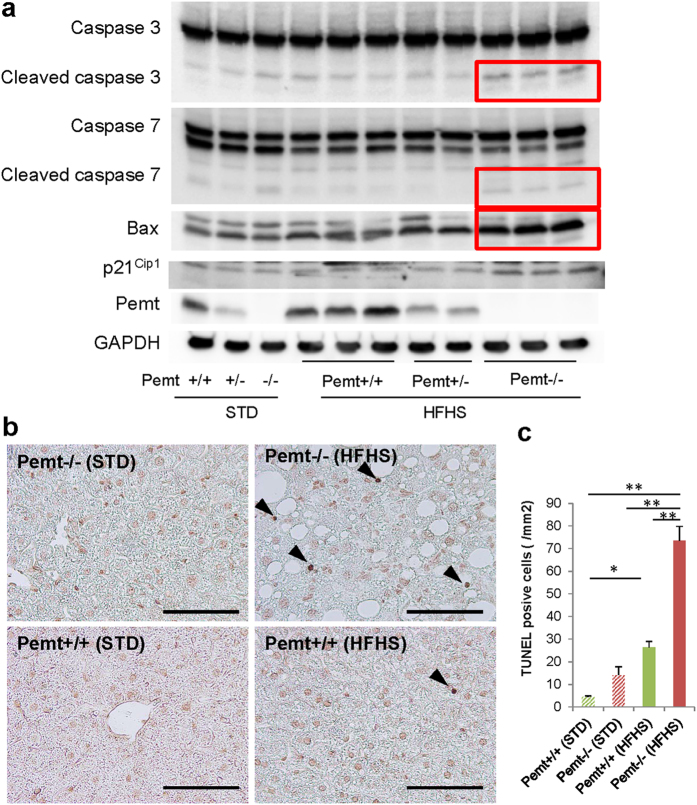
Apoptosis of hepatocytes in Pemt+/+, Pemt+/− and Pemt−/− mice at 25 weeks of age. (**a**) Western blot analyses of liver tissue samples for pro-apoptotic molecules, caspases 3 and 7, Bax, and p21^Cip1^. Cleaved caspase 3 (17 and 19 kDa), cleaved caspase 7 (20 kDa) and Bax prominently up-regulate in Pemt−/− mice fed high fat-high sucrose diet (red squares). (**b**) TUNEL staining of the liver. TUNEL positive cells are shown by arrow heads. **c**. TUNEL-positive apoptotic cells/mm^2^. The apoptotic cells increase in Pemt−/− mice compared to Pemt+/+ mice under high fat-high sucrose diet. All data are presented as mean ± S.E. n = 3–6. *p < 0.05, **p < 0.01.

**Figure 5:**
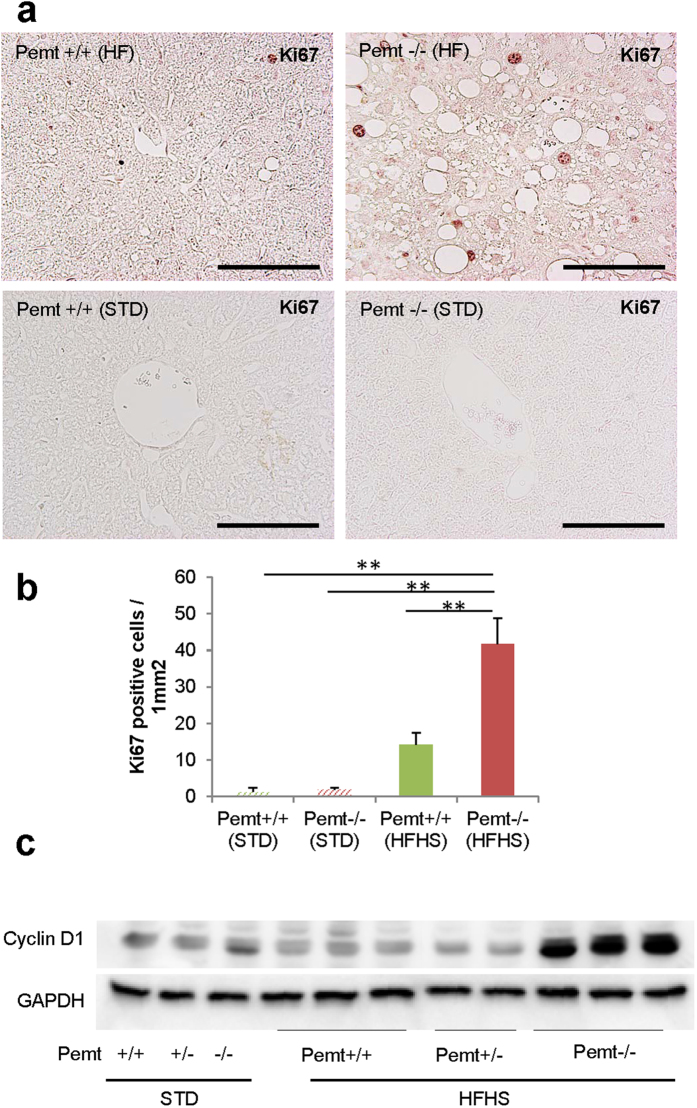
Proliferation of hepatocytes in Pemt+/+, Pemt+/− and Pemt−/− mice at 25 weeks of age. (**a**) Ki67 immunohistochemical staining of liver tissues. (**b**) Ki67 positive cells/mm^2^. Proliferating cells increase in Pemt−/− mice under high-high sucrose diet. All data are presented as mean ± SE. n = 2–6. *p < 0.05, **p < 0.01. (**c**) Western blotting analyses of cyclin D1 in liver tissues.

In previous reports, apoptosis of hepatocytes was enhanced and regeneration impaired in steatohepatitis, we next investigated the status of apoptosis and cell proliferation in Pemt−/− mice fed HFHS chow. In Pemt−/− mice fed HFHS chow, the expression of cleaved caspase 3, 7 and Bax protein prominently increased (Fig. 4a), and the number of TUNEL-positive apoptotic cells significantly increased in Pemt−/− mice compared to Pemt+/+ mice fed HFHS chow (Fig. 4b,c). To investigate the major inducers for the apoptosis of Pemt deficient hapatocytes under HFHS chow, we treated H-4-II-E-C3 cells with insulin, palmitate, high glucose and IL-6 in the presence of shRNA-CTRL or shRNA-Pemt (Supplementary Fig. 5). Insulin-induced up-regulation of pro-apoptotic molecules such as cleaved caspases 3 and 7 was suppressed and the activation of anti-apoptotic molecule, *i.e*. phosphorylation of Akt enhanced by the knockdown of Pemt. However, palmitate-induced cleaved caspases 3 and 7 were not reduced and rather increased in shPemt-treated H-4-II-E-C3 cell. High glucose and IL-6 did not affect the expression of cleaved caspase 3 and 7, and phosphorylation of Akt. Although prominent apoptosis in hepatocytes was characteristic features in the liver of Pemt−/− mice fed HFHS chow, the number of Ki67-positive hepatocyte increased in Pemt−/− mice compared to Pemt+/+ mice fed HFHS diet (Fig. 5a,b). The protein expression of cyclin D1 also highly up-reglated in Pemt−/− mice fed HFHS diet (Fig. 5c).

### Pemt reduces p53-regulated genes by interacting with clathrin heavy chain (CHC)

**Figure 6:**
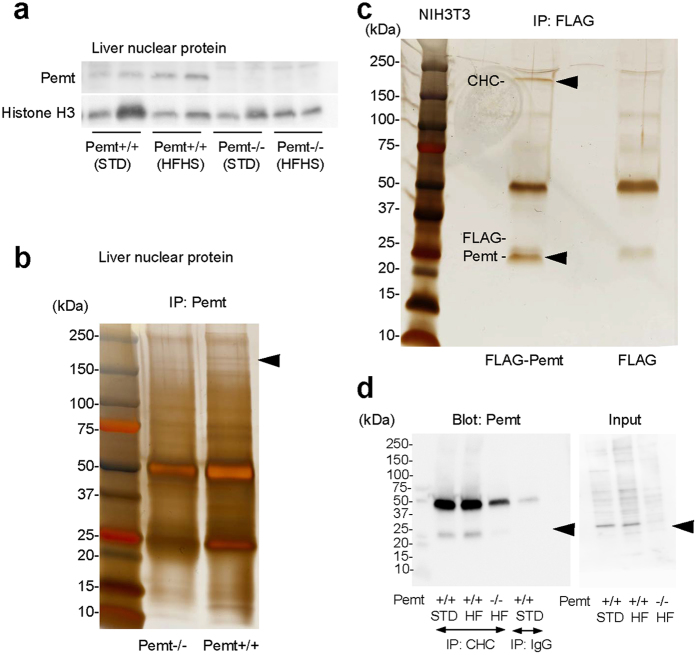
Localization of Pemt in nucleus and interaction with clathrin heavy chain (CHC). (**a**) Western blot analyses of liver nuclear protein. (**b**) Immunoprecipitation of the nuclear protein from Pemt+/+ and Pemt−/− mice. Nuclear samples immunoprecipitation with anti-Pemt antibody were subjected to SDS-PAGE. CHC was identified as an interacting molecule with Pemt by in-gel digestion with trypsin and LC-MS/MS analyses. (**c**) Immunoprecipitation of NIH3T3 cells overexpressing FLAG-Pemt. Cell lysates were immunoprecipitated with anti-FLAG antibody and analyzed by SDS-PAGE. CHC is indicated by arrow head. (**d**) Immunoprecipitation using nuclear protein of liver samples with anti-CHC antibodies. Immunoprecipitaes were blotted with anti-Pemt antibodies and the binding of Pemt with CHC are confirmed. STD; standard diets, HFHS; high fat-high sucrose diets.

Although Pemt has been demonstrated to localize mainly in ER and mitochondria, the evidences of prominent apoptotic and proliferative activities of hepatocytes in Pemt−/− mice fed HFHS chow prompted us to further investigate the subcellular localization of Pemt in nuclei of hepatocyte in Pemt+/+ mice and H-4-II-E-C3 cell. In the nuclear extracts of liver tissues, the presence of Pemt was confirmed by Western blot analyses (Fig. 6a). The nuclear extracts from Pemt+/+ and Pemt−/− mice were further subjected to the immunoprecipitation with anti-Pemt antibody. In SDS-PAGE, we found ~170 kDa band in Pemt+/+ but not in Pemt−/− mice (Fig. 6b, arrow), and CHC was identified by in-gel digestion with trypsin and LC-MS/MS analyses. We next confirmed the complex formation of FLAG-Pemt and CHC by the immunoprecipitation using FLAG-Pemt-transfected NIH3T3 cells and anti-FLAG antibody (Fig. 6c). Furthermore, Pemt-CHC complex was confirmed by immunoprecipitation using nuclear lysates isolated from the liver tissues and anti-CHC antibody (Fig. 6d). CHC is consisted of β propeller repeat (amino acid residues 1–330), α superhelix linker (residues 331–494), and multiple α helical repeat (543–1576). Enari *et al.* reported that p53 binds to CHC at the C-terminal region containing clathrin light chain (CLC)-binding and trimerization domains. To identify Pemt-binding domain of CHC, we generated 3 segments of CHC protein tagged with N-terminal myc epitope; myc-CHC (232–340), myc-CHC (1074–1406), and myc-CHC (1267–1513). After co-expression of FLAG-Pemt and myc-CHC in NIH3T3 cells, they were immunoprecipitated by anti-FLAG antibody and blotted with anti-myc antibody. Pemt interacts with p53-binding (1074–1406) and clathrin light chain-binding (1267–1513) domains (Supplementary Fig. 6a). We further confirmed the binding between p53 and Pemt in the nuclear proteins derived from NIH3T3 cells and liver tissues of Pemt+/+ mice (Supplementary Fig. 6b,c).

**Figure 7:**
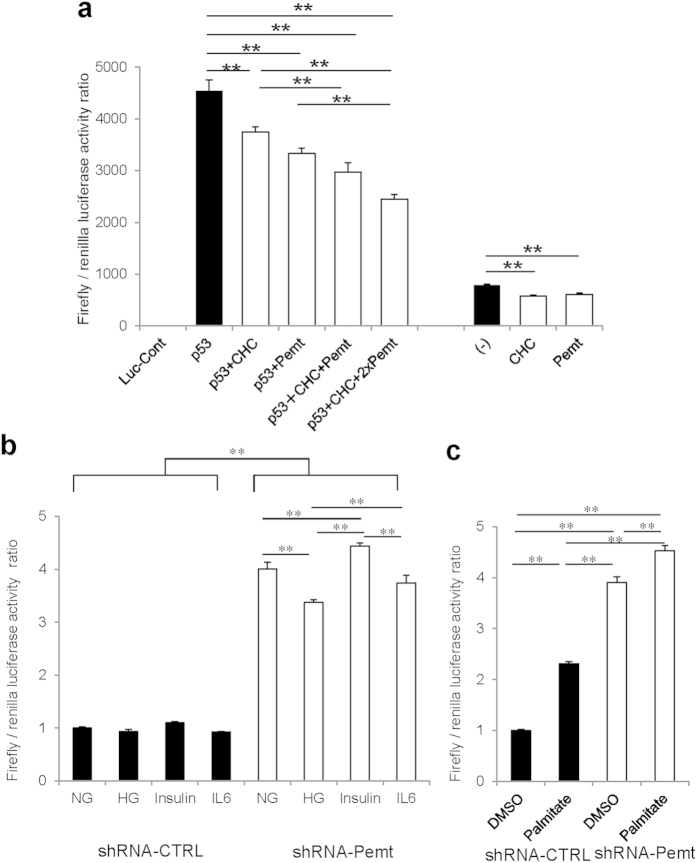
Luciferase reporter gene assay. (**a**) Luciferase reporter gene assay using COS-7 cells transfected with p53-Luc *Cis*-reporter Plasmid. COS-7 cells were transiently transfected with Pathdirect p53-Luc *Cis*-reporter Plasmid. For negative control vector, pCIS-CK supplied by manufactures was used. FLAG-Pemt-HA (Pemt), myc-CHC (CHC) and p53-HA (p53) plasmids were co-expressed in COS-7 cells. Overexression of Pemt and CHC additively reduced p53-luciferase activities. Data are means ± SE. n = 5 in all groups. **P < 0.01. (**b**,**c**) Luciferase reporter gene assay using H-4-II-E-C-3 cells treated with shRNA-CTRL or shRNA-Pemt. H-4-II-E-C-3 cells were transiently transfected with Pathdirect p53-Luc *Cis*-reporter Plasmid and treated with 25 mM high glucose (HG), 100 nM insulin, 20 ng/ml IL-6, or 250 μM Palmitate for 24 hr. In H-4-II-E-C-3 cells, the treatments with shRNA-Pemt increase p53-luciferase activities compared to shRNA-CTRL. Palmitate and insulin stimulate p53-luciferase activities in shRNA-Pemt-treated H-4-II-E-C-3 cells. Data are means ± SE. n = 5 in all groups. **P < 0.01.

p53 protein is well-known as a transcription factor, which binds to p53-responsive element of promoter region of various p53 target genes. To evaluate whether Pemt and CHC have inhibitory effects on p53-driven transcription, we performed luciferase assay using p53-Luc Cis-Reporter Plasmid and COS-7 cells. Both basal and induced Firefly/Renilla luciferase activity ratio by p53 overexpression was suppressed by gene delivery of either Pemt or CHC genes (Fig. 7a). When both of Pemt and CHC genes were transfected, the additive suppression of p53-luciferase activity was observed. In contrast, the knockdown of Pemt in H-4-II-E-C3 cells using lentiviral Pemt shRNA vector (shRNA-Pemt) significantly increased the luciferase activity compared with Non-Target shRNA transfected cells (shRNA-CTRL)(Fig. 7b,c). Palmitate and insulin are established stimulator for recruitment of p53 to p53-responsive element under obese states and they increased p53-luciferase activities in shRNA-Pemt-treated H-4-II-E-C3 cells, while high glucose condition and IL-6 stimulation decreased luciferase activities (Fig. 7b,c). Taken together, Pemt insufficiency enhanced transcriptional activities of p53-target genes, expression of cleaved caspases 3, 7, and Bax, and subsequent apoptosis of hepatocytes.

### The crosstalk between Pemt and CHC in the regulation of their expression

The knockdown of Pemt increased the expression of CHC in H-4-II-E-C3 cell (Supplementary Fig. 5). The treatment of insulin, palmitate, high glucose and IL-6 increased the expression of CHC under the presence of shRNA-Pemt (Supplementary Fig. 5). We next evaluate the regulation of expression via crosstalk between Pemt and CHC by co-transfection of myc-CHC and FLAG-Pemt plasmid vectors into COS-7 cells. Under the constant concentration of FLAG-Pemt vector, Pemt expression increased along with increased doses of myc-CHC vector (Supplementary Fig. 7a). Conversely, the transfection of incremental concentrations of FLAG-Pemt vector suppressed the expression of CHC in a dose-dependent manner (Supplementary Fig. 7b). In the liver of Pemt−/− mice, CHC expression increased compared to Pemt+/+, and HFHS diet further increased CHC expression in the liver compared to that expression in STD died-fed mice (Supplementary Fig. 7c). The expression of CHC in liver nuclear extracts was also increased under HFHS chow and insulin administration (Supplementary Fig. 7d). Taken together, it suggested that Pemt suppressed the CHC expression, and CHC promoted Pemt expression.

### Pemt deficiency promotes DNA methylation and reduces mRNA expression of Fbxo31 and HNF4α

**Figure 8:**
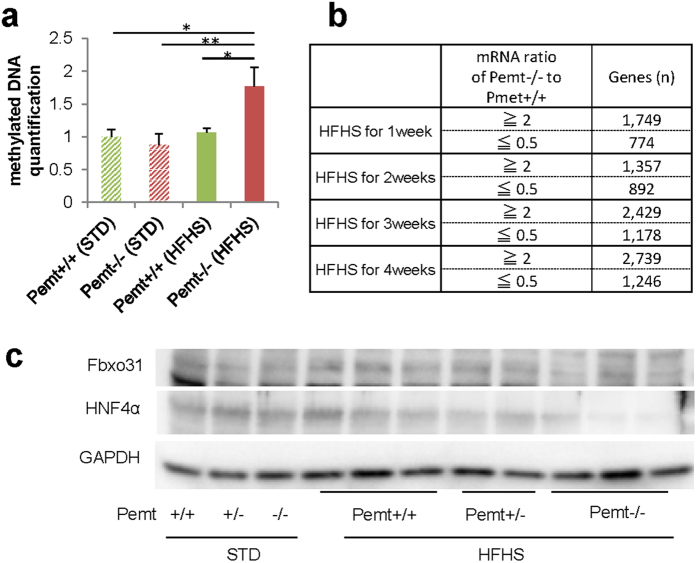
Methylation analysis of liver in Pemt+/+ and Pemt−/− mice under high fat-high sucrose (HFHS) diet. (**a**) Quantification of DNA methylation using Imprint Methylation DNA Quantification Kit. In Pemt−/− under HFHS diet, methylated DNA increased compared to Pemt+/+ mice. (**b**) mRNA expression profiling by DNA chip analysis. The numbers of gene with RNA expression ratio (Pemt−/− to Pemt+/+) over 2 or under 0.5 are shown at 1, 2, 3 and 4 weeks after the feeding with HFHS diet. (**c**) Western blot analyses of F-box protein 31 (Fbxo31) and hepatocyte nuclear factor 4 alpha (HNF4α) in liver samples from Pemt+/+, Pemt+/− and Pemt−/− mice.

Pemt is methyltransferase, which converts phosphatidylethanolamine to phosphatidylcholine using S-adenosylmethionine (SAM) as a methyl donor and the knockout of Pemt may be linked to increased methyl donor, enhanced methylation of DNA and alterations in mRNA expressions. In Pemt−/− mice under HFHS diet, the ELISA quantification of methylation demonstrated the increase in total methylation of genomic DNA (Fig. 8a). We further performed sequencing-based DNA methylation analysis by Genome Analyzer IIx and mRNA expression profiling by DNA chip analysis. The genes with RNA expression ratio (Pemt−/− to Pemt+/+) over 2 or under 0.5 were extracted at 1, 2, 3, and 4 weeks after the feeding with HFHS chow (Fig. 8b). By integrating DNA methylation and mRNA expression data, we further selected the 60 genes with hypermethylated genomic DNA and reduced mRNA expression in Pemt−/− mice compared to Pemt+/+ fed HFHS for 4 weeks. Finally, sixteen genes with dowregulated mRNA expression at 4 weeks compared with baseline were chosen (Supplementary Table 2). Among them, F-box only protein 31 (Fbxo31) is involved in cyclin D1 degradation by direct interaction^26^, and hepatocyte nuclear factor 4 is essential for differentiation of hepatocyte and negatively regulates cyclin D1^27^. The methylation sequence depth of Fbxo31 and HNF4α was shown in Supplementary Fig. 8 and 9, respectively. The protein expression of F-box protein 31 (Fbxo31) and hepatocyte nuclear factor 4 alpha (HNF4α) indeed decreased in Pemt−/− fed HFHS compared with Pemt+/+ mice (Fig. 8c).

### PEMT mRNA expression in liver of NASH patients is reduced

We recruited 34 patients with nonalchoholic fatty liver disease underwent liver biopsy from June 2009 to December 2012. Histological diagnosis was performed according to Matteoni’s classification^28^. Among 34 patients, 9 cases were diagnosis as simple steatosis (SS), and 25 cases as nonalchoholic steatohepatitis (NASH). Asparteate aminotransferase (AST), immunoreactive insulin (IRI), and homeostasis model assessment-Insulin resistance (HOMA-IR) was higher than these in NASH patients (p = 0.014, p = 0.013, respectively). *PEMT* mRNA expression in liver of NASH patients was significantly lower than in NAFLD patients (p = 0.042) (Supplementary Table 3 and 4). *PEMT* mRNA expression significantly correlated with platelet counts, which are known to decline as fibrosis progresses (Supplementary Table 5). Finally, lower quartile of PEMT mRNA (Q2) demonstrated lower BMI and platelet counts (Supplementary Fig. 10), suggesting lower expression of PEMT mRNA is related to the development of lean NASH.

## Discussion

Although the disruption of Pemt gene revealed the minimal phenotype in the liver tissue of the mice fed normal chow^29^, Pemt−/− mice fed choline-deficient diet developed steatohepatitis and liver failure after 3 days^30^. The PC/PE ratio is a key regulator of cell membrane integrity and a disturbance in the ratio plays an important role in the progression of steatosis to steatohapatitis under choline-deficient diet^31^. Pemt−/− mice were protected from insulin resistance and obesity under high-fat diet^23^. The deficiency in choline biosynthesis seemed to provide a beneficial effect in diabetes and obesity, since choline supplementation promotes the hepatic insulin resistance in Pemt−/− mice fed high-fat diet^32^. Although the liver is mainly involved in determining their phenotype and the activity of Pemt is relatively lower in adipose tissues, the conversion of PE to PC in adipocytes appears to be important for the stabilization of lipid droplets and normal fat distribution^33^. Pemt−/− mice fed high-fat diet for 10 weeks developed steatosis in previous report, although the mice were protected from obesity and diabetes^23^. The hepatic steatosis is caused by the inability of adipocytes for the maintenance of lipid droplets and the requirement of Pemt in the secretion of apoB100-containing VLDLs from the liver^34^. In contrast to the series of the investigation, Fu *et al.* reported the rise of PC/PE ratio and upregulation of Pemt in hepatic endoplasmic reticulum (ER) in obesity, which resulted in reduced calcium transport activity of sarco/endoplasmic reticulum calcium ATPase (SERCA) and enhanced ER stress^35^. The suppression of Pemt in the liver by adenovirally expressed short hairpin RNA (shRNA) for Pemt resulted in reduction of PC/PE ratio, improvement of SERCA activity, amelioration of ER stress associated with improvement of hepatic steatosis and glucose homeostasis^35^. The discrepancy of the results may be derived from differences in experimental design, systemic deletion *v.s.* liver-specific knockdown of Pemt gene, and also from short period of observations.

In current experiment, we extended the observation period of Pemt−/− mice fed HFHS chow to 90 weeks and they were completely protected from obesity and diabetes. However, Pemt−/− mice fed HFHS chow developed prominent steatohepatitis and multiple liver tumors. Pemt−/− mice fed HFHS chow were further characterized with enhanced apoptosis and prominent proliferation of hepatocytes. The long-term course of Pemt−/− mice fed HFHS completely corresponded to the clinical pictures and phenotypes of lean NASH in human. The overaccumulation of free fatty acid causes a distinct type of apoptosis, *i.e.* ‘lipoapoptosis’ in pancreatic β-cells and such lipotoxicity is an important in the pathogenesis of type 2 diabetes in obesity and metabolic syndrome^36^,^37^. The process of lipoapoptosis has been one of the important contributors for the progression of NASH^38^,^39^ and tumor formation^40^. Fatty liver is considered as one of the major risk factors for the impaired liver regeneration and replication^41^, however, enhanced proliferation activities were demonstrated in Pemt−/− mice fed HFHS diet by increased number of Ki67-positive hepatocytes and highly upregulated expression of cyclin D1. Thus, we further investigated the mechanism for the enhancement of the apoptosis and cell proliferation in the liver of Pemt−/− mice fed HFHS diet. The p53 protein is a transcription factor that activates various genes and responsible for growth arrest and apoptosis in response to various DNA damage. Clathrin self-assembles into a coat around vesicles filled with cargo such as nutrients, hormones, and proteins destined for degradation^42^,^43^. Although CHC was identified as a cytosolic protein regulating endocytosis, it is present in nuclei, binds to p53 and functions as coactivator for p53^44^,^45^. Monomeric CHC (833–1406) but not trimeric CHC had a higher ability to transactivate the p53^46^ and Asn1288 is critical for the binding to p53^47^. In current study, we demonstrated that Pemt interacts with p53-binding (1074–1406) and clathrin light chain-binding (1267–1513) domains and inhibit the transactivation of p53. Since cleaved caspases 3 and 7, Bax, and p21^Cip1^ increased Pemt−/− mice fed HFHS diet, p53 drives responses against the cellular stress by the overaccumulation of free fatty acid in the liver through cell death and senescence^48^,^49^,^50^. Prominent apoptosis in the liver of Pemt−/− mice fed HFHS diet is explained by transactivation of p53 but there must be primary cause for the enhanced proliferation of hepatocytes. The sustained lipoapoptosis promotes the inflammation^36^,^38^ and increased expression of CHC also contribute the such process, since CHC is essential for the TNF-α-induced inflammatory signaling pathways^51^. We further performed genome-wide sequencing-based DNA methylation analysis by Genome Analyzer IIx and mRNA expression profiling by DNA chip analysis. We finally found out that genomic DNA methylation of Fbxo31 and HNF4α is enhanced and their mRNA expression is reduced. Since Fbxo31 is involved in cyclin D1 degradation by direct interaction^26^ and HNF4α is essential for differentiation of hepatocyte and negatively regulates cyclin D1^27^, down-regulation of Fbxo31 and HNF4α is responsible for the upregulation of cyclin D1 in hepatocytes in Pemt−/− mice fed HFHS diet.

The current investigation is suffered from several limitations as follow. Although the deletion of *Pemt* in mice fed with high-fat diet strongly supports the idea that the loss of *Pemt* is major driver for liver fibrosis, the prospective cohort study instead of cross-sectional study design is required to prove the causality in the patients with SS and NASH. In addition, the data of *PEMT* expression and activity is still limited in viral and autoimmune hepatitis and other diseases such as lipodystrophic patients associated with severe fatty liver and loss of adipose tissues. Similarly, we should investigate the expression and activity of *Pemt* in the animal models with “lean NASH” such as MCD-diet fed mice and *Pten*-deficient^52^ mice in future studies. In *leptin*−/− C57BL6 mice, the specific knockdown of *Pemt* mRNA by adenovirally expressed *Pemt*-shRNA in liver ameliorated fatty liver disease^35^ and it was contradict report compared with studies by us and others^23^. Thus, the tissue-specific deletion of *Pemt* in adipose or liver tissues would further elucidate the role of *Pemt* in pathogenesis of “lean NASH” whether hepatic and extrahepatic phenotypes are necessary or sufficient for the liver outcome. Finally, major effector molecules of *Pemt* are still unexplored since rescue experiments using CDK4-cyclin D1 inhibitors and p53 inhibitors were not performed in current investigation.

The deficiency of Pemt and high-fat diet in mouse model demonstrated the phenotypes resemble to the clinical features of the patients with lean NASH. Actually, mRNA expression of PEMT is lower in the patients with NASH compared with those with SS. In addition, lower quartiles of PEMT mRNA demonstrated lower BMI and platelet counts, suggesting lower expression of PEMT is critically linked to the pathogenesis of lean NASH. In previous study, the carriers with Val75Met variant of PEMT gene demonstrated impaired PEMT activity, more frequent in NASH patients than healthy volunteers, lower BMI and more non-obese patients^53^, all supporting our observation. In this clinical entity of lean NASH, the restriction of fat contents and supplementation of choline is required and measurement of PEMT activity is beneficial to define the clinical entity of lean NASH. Further clinical investigations of lean NASH patients with genotyping of PEMT and measurement of PEMT activities are required. In addition, pharmacological PEMT inhibition may be beneficial in the treatment of obesity and type 2 diabetes with concomitant diet therapy with fat restriction or use of lipase inhibitors (Supplementary Fig. 18).

## Methods

### Animals

Male Pemt−/−, Pemt+/− and Pemt+/+ mice were housed in cages and maintained on a 12-hour light-dark cycle. They were fed standard (MF, Oriental Yeast, Co., Ltd) and high fat-high sucrose (HFHS) (D12331; Research Diet) chow and sacrificed at 25, 60 and 90 weeks of age. All animal experiments were approved by the Animal Care and Use Committee of the Department of Animal Resources, Advanced Science Research Center, Okayama University. All animal experiments were carried out in accordance with the approved guidelines.

### Cell Culture

H-4-II-E-C3 cells (ECACC) were cultured in MEME (Minimum Essential Medium Eagle) containing 2 mM glutamine, 1% non-essential amino acids and 10% fetal bovine serum. NIH3T3 cells (ECACC) were cultured in DMEM (Dulbecco’s Modified Eagle’s Medium) containing 2 mM glutamine and 10% calf serum. COS-7 cells (ATCC) were cultured in DMEM with 10% feral bovine serum. H-4-II-E-C3 cells were transfected with 5 MOI (multiplicity of infection) of MISSION shRNA lentivirus transduction particles for Pemt (NM_008819) and Non-Target shRNA control lentivirus transduction particles, and they were further treated with high glucose (25 mM), 100 nM insulin (I9278, SIGMA), 250 μM palmitate (P0500, SIGMA), and 20 ng/ml IL-6 (I1395, SIGMA). For the preparation of palmitate stock solution, 500 mM palmitate in DMSO was diluted to 5 mM with 5% BSA-contained PBS, and sonicated at 50 °C until complete dissolution. As a negative control, PBS containing 1% DMSO and 5% BSA was used.

### Plasmids

Full coding cDNA of Pemt was amplified by using PCR primers (Pemt-*EcoR*I-F and Pemt-*Xba*I-R) and ligated to *EcoR*I-*Xba*I site of p3xFLAG CMV expression vector (SIGMA) (FLAG-Pemt). Oligodeoxynucleotides coding myc-tag was annealed and ligated to *BamH*I-*EcoR*I site of pcDNA3 (Invitrogen) (myc). Full and partial coding cDNAs of CHC were prepared by PCR primer sets, CHC1-*No*tI-F and CHC1-*Apa*I-R for CHC^232–340^, CHC3-*No*tI-F and CHC3-*Apa*I-R for CHC^1074–1406^, CHC4-*No*tI-F and CHC4-*Apa*I-R for CHC^1267–1513^, and CHC2-*No*tI-F and CHC2-*Apa*I-R for full length CHC, and they ligated into *No*tI-*Apa*I site of pcDNA3 (Invitrogen) (myc-CHC232–340, Myc-CHC1074–1406, Myc-CHC1267–1513 and Myc-CHC). Coding cDNA of p53 tagged with C-terminal hemagglutinin epitope (HA) was prepared by PCR primers (p53-HA-F and p53-HA-R) and ligated to *Hin*dIII-*XhoI* site of pcDNA3.1/V5-His-TOPO (Invitrogen) (p53-HA). Coding cDNA of Pemt tagged with N-terminal FLAG and C-terminal hemagglutinin (HA) epitopes were prepared by primers (FLAG-Pemt-HA-F and FLAG-Pemt-HA-R) and ligated to *Hin*dIII-*XhoI* site of pcDNA3.1/V5-His-TOPO (FLAG-Pemt-HA). Primers are shown in Supplementary Table 1.

### Luciferase reporter gene assay

COS-7 or H-4-II-E-C3 cells were transiently transfected with FLAG-Pemt-HA, Myc-CHC, p53-HA and Pathdirect p53-Luc *Cis*-Reporter Plasmid (Stragene) using Lipofectamine LTX Reagent (invitorogen) following manufacturer’s protocol. Luciferase activities were measured with a dual-luciferase assay system and GloMax 20/20n Luminometer (Promega). pCIS-CK supplied in Pathdirect *Cis*-Reporting System was used as a negative control.

### Western blot analysis

Liver tissues were excised and homogenized with lysis buffer (20 mM Tris-HCl, pH 7.4, 100 mM NaCl, 10 mM benzamidine-HC, 10 mM ε-amino-*n*-caproic acid, 2 mM phenylmethylsulfonyl fluoride and 1% Triton X-100). After centrifugation at 14,000 rpm for 30 min at 4 °C, the supernatants were collected for further analyses. Total lysate of H-4-II-E-C3 cells were also collected and nuclear proteins were purified by Nuclear Extract Kit (Active Motif). Equal amount of protein was subjected to SDS-PAGE under reducing conditions, and electroblotted onto Hybond P polyvinylidene fluoride membranes (GE Healthcare Life Sciences). The membranes were immersed in blocking solution containing 5% nonfat dry milk and Tris-buffered saline with Tween-20 (0.05% Tween-20, 20 mM Tris-HCl, and 150 mM NaCl, pH 7.6). Then, the membranes were incubated with primary antibodies; rabbit anti-phospho-Akt (Ser473), anti-Akt, anti-Caspase-3, anti-Caspase-7, anti-Bax, anti-DYKDDDK Tag, anti-TGF-β, anti-cyclin D1, rabbit anti-GAPDH (14C10), anti-ATF-4 (D4B8), mouse monoclonal anti-p53 (1C12) (Cell Signaling Technology), rabbit anti-α smooth muscle actin, anti-FBXO31, anti-Histone H3, anti-clathrin heavy chain antibody (abcam), rabbit polyclonal anti-p21 (H-164), mouse monoclonal anti-p53 (2B2.68) (Santa Cruz Biotechnology) and mouse monoclonal anti-FLAG2 M2, rabbit polyclonal anti-PEMT (SIGMA), and anti-PEMT antibody (novusbio). They were then incubated with anti-rabbit or anti-goat IgG conjugated with horseradish peroxidase (GE Healthcare Life Sciences). The blots were washed three times with Tris-buffered saline with Tween-20, immersed in ECL Plus Western Blotting Detection Reagents (GE Healthcare Life Sciences), and the chemiluminescence was analyzed using the LAS-3000 mini instrument (FUJIFILM).

### LC-MS/MS (high performance liquid chromatography-tandem mass spectrometry)

We performed immunoprecipitation with liver nuclear proteins of Pemt−/−, Pemt+/+ liver using anti-Pemt antibodies. The bands, visible only in Pemt+/+ mice, were excised and in-gel-digested with trypsin and analyzed with LC-MS/MS.

### Morphological studies

Liver tissue specimens were fixed in 10% formaldehyde and embedded in paraffin and 4 μm-thick sections were prepared. For antigen retrieval, they were deparaffinized, rehydrated and pretreated by microwave for 10 minutes in Target Retrieval Solution (DAKO). Nonspecific binding was blocked by incubation in 10% goat or rabbit serum for 30 min. The tissue sections were incubated with rabbit monoclonal anti-Ki-67 (D3B5) antibody (Cell signaling Technologies), anti-Cytokeratin 19 [EP1580Y], rabbit polyclonal anti-α fetoprotein antibody (abcam), rat monoclonal anti-F4/80 [CI:A3-1] antibody (abcam) at 4 °C overnight. After being washed in PBS, they were incubated with a biotinylated secondary antibody and VECTASTAIN ABC Standard Kit (Vector Laboratories, Burlingame, CA). Immunochemical staining was performed with the ImmPACT DAB SUBSTRATE (Vector Laboratories). The tissues were stained with periodic acid-Schiff (PAS) and Masson-Trichrome, and apoptotic cells in liver tissues were detected by DeadEnd^TM^ Colorimetric TUNEL system (Promega).

### Methylaytion analysis of liver genomic DNA and DNA microarray analysis

Liver DNAs were extracted using DNeasy Blood & Tissue Kit (n = 3–4) from Pemt−/− and Pemt+/+ mice fed with HFHS or STD chow for 25 weeks. One hundred ng of DNA was subjected to Imprint Methylation DNA Quantification Kit (SIGMA) to measure the methylation of DNA. Pemt−/− (n = 4) and Pemt+/+ (n = 4) mice were fed with HFHS chow and liver samples were obtained at 1, 2, 3 and 4 weeks. Methylated DNA was enriched with EpiXplore Methylated DNA Enrichment Kit (Clontech) according to manufacturer’s protocol. Genomic DNA libraries was prepared by TruSeq ChIP Sample Preparation Kit (Illumina) and sequenced by Genome Analyzer IIx. For DNA microarray, total RNAs were extracted by using RNeasy Midi Kit (QIAGEN) and subjected to GeneChip Expression Assay (Affymetrix). The whole raw and processed data are freely available in the Gene Expression Omnibus (GEO) under the accession number GSE67791, GSE67792 and GSE67793.

### PEMT mRNA expression in the patients with non-alcoholic liver disease

Japanese patients with biopsy-proven non-alcoholic fatty liver disease admitted to Department of Gastroenterology, Okayama University Hospital from June 2009 to December 2012 were recruited to the current investigation. Liver histology was evaluated according to Matteoni’s classifications^28^. For the quantitative real time PCR analysis, cDNAs synthesized from 2 μg of total RNA isolated from liver specimens were amplified in the presence of primers and TaqMan Minor Groove Binder Probes (TaqMan Gene Expression Assays; Applied Biosystems, Carlsbad, CA) using a StepOnePlus Real Time PCR System. The relative abundance of PEMT mRNA (Hs00540979_m1, Applied Biosystems) was standardized using 18S mRNA (Hs99999901_s1) as the internal control.

### Statistics

Data are expressed as the means ± standard error (SE) and analyzed by the unpaired Student’s *t* test in the comparison of two groups and two-way analysis of variance (ANOVA) in the comparison of more than three groups. P < 0.05 was regarded as statistically significant. The data were analyzed with IBM SPSS Statistics Ver. 22.0 (IBM).

### Study approval

All animal experiments were approved by the Animal Care and Use Committee of the Department of Animal Resources, Advanced Science Research Center, Okayama University. The human study was conducted in accordance with the ethical principles of the Declaration of Helsinki and was approved by the ethical committee of Okayama University Graduate School of Medicine, Dentistry, and Pharmaceutical Sciences (Registration numbers 1782, 1878, 1957). Written informed consent was received from participants prior to inclusion in the study.

## Additional Information

**How to cite this article**: Nakatsuka, A. *et al.* Insufficiency of phosphatidylethanolamine *N*-methyltransferase is risk for lean non-alcoholic steatohepatitis. *Sci. Rep.* **6**, 21721; doi: 10.1038/srep21721 (2016).

## Supplementary Material

Supplemental Figure 1-18, Supplemental Table 1-5
