# Supplementary material for: Insufficiency of phosphatidylethanolamine N-methyltransferase is risk for lean non-alcoholic steatohepatitis
Source: Sci Rep. 2016 Feb 17;6:21721. doi: 10.1038/srep21721 (PMC4756298; doi:10.1038/srep21721)
Supplement: Supplemental Figure 1-18, Supplemental Table 1-5 [file srep21721-s1.pdf]

Insufficiency of phosphatidylethanolamine *N*-methyltransferase is risk for lean non-alcoholic steatohepatitis

Atsuko Nakatsuka[1], Makoto Matsuyama[2], Satoshi Yamaguchi[1], Akihiro Katayama[1], Jun Eguchi[1], Kazutoshi Murakami[1, 3], Sanae Teshigawara[1], Daisuke Ogawa[4], Nozomu Wada[5], Tetsuya Yasunaka[5], Fusao Ikeda[5], Akinobu Takaki[5], Eijiro Watanabe[6], and Jun Wada[1]

[1]*Department of Medicine and Clinical Science, Okayama University Graduate School of Medicine, Dentistry and Pharmaceutical Sciences, Kita-ku, Okayama 700-8558, Japan*

[2]*Shigei Medical Research Institute, Minami-ku, Okayama 701-0202, Japan*

[3]*Department of General Medicine, Okayama University Graduate School of Medicine, Dentistry and Pharmaceutical Sciences, Kita-ku, Okayama 700-8558, Japan*

[4]Department of Diabetic Nephropathy, Okayama University Graduate School of Medicine, Dentistry and Pharmaceutical Sciences, Kita-ku, Okayama 700-8558, Japan

[5]*Department of Gastroenterology and Hepatology, Okayama University Graduate School of Medicine, Dentistry and Pharmaceutical Sciences, Kita-ku, Okayama 700-8558, Japan*

[6]*Dainippon Sumitomo Pharma, 2-6-8 Doshomachi, Chuo-Ku, Osaka 541-0045, Japan*

Supplemental Figure legends

Supplemental Figure 1. Body weight and glucose tolerance in Pemt+/+, Pemt+/-, Pemt-/- mice under standard (STD) and high fat-high sucrose (HFHS) diets. a. Body weight of Pemt+/+, Pemt+/-, Pemt-/- mice under STD and HFHS diets. n=8-26. **b** and **c.** Epididymal fat (**b**) and liver weight (**c**) of Pemt+/+, Pemt+/- and Pemt-/- mice at 25 weeks of age. n=5-9. **d-g.** Glucose tolerance test at 12 weeks of age (1.5 mg/kg BW *i.p.*). n=6-13. **h** and **i.** Insulin tolerance test at 12 weeks of age (0.75 IU/kg for STD and 1.0 IU/kg BW for HFHS *i.p.*). n = 5-9. All values are presented as mean ± SE. *P < 0.05, **P < 0.01 versus Pemt+/+ mice.

Supplemental Figure 2. Metabolic data of Pemt+/+, Pemt+/-, Pemt-/- mice under standard (STD) and high fat-high sucrose (HFHS) diet at 25 weeks of age. a. *ad Librium* Blood glucose. **b.** Fasting blood glucose. **c.** Serum total cholesterol. **d.** Serum triglyceride. **e.** Serum adiponectin levels. **f.** Serum leptin levels. All data are presented as mean ± SE. n =5-6. *P < 0.05, **P < 0.01.

Supplemental Figure 3. Macrophage infiltration and expression of fibrogenic molecules in the liver under high fat-high sucrose (HFHS) diets. a. Immunostaining of F4/80**.** Bar=100 μm. **b.** Western blot analyses of liver samples.

Supplemental Figure 4. Electron micrograph of hepatocytes in Pemt+/+ and Pemt-/- fed with high fat-high sucrose (HFHS) diets at 25 weeks of age. a. Marked deposition of glycogen granules (asterisk) in Pemt+/+ mice under HFHS diet. **b.** Apparently large lipid droplets (L) and decreased glycogen granules in Pemt-/- mice under HFHS diet. **c.**

Prominent rough endoplasmic reticulum (ER) with lamellar structure in Pemt+/+ mice under HFHS diet. **d.** Numerous large mitochondria (M) and round-shaped smooth ER in Pemt-/- mice.

Supplemental Figure 5. Western blotting analyses of H-4-II-E-C-3 cells treated with shRNA-CTRL or shRNA-Pemt. Insulin-induced up-regulation of pro-apoptotic cleaved caspases 3 and 7 are suppressed and anti-apoptotic Akt phosphorylation is enhanced by knockdown of Pemt. Palmitate-induced pro-apoptotic molecules, cleaved caspases 3 and 7, increased in shRNA-PEMT-treated cells. NG; normal glucose (5 mM), HG; high glucose (25 mM).

Supplemental Figure 6. Interaction of Pemt with clathrin heavy chain (CHC) and Pemt-CHC-p53 tri-complex formation. a. Immunoprecipitation using COS-7 cells expressing FLAG-Pemt and myc-CHC. FLAG-Pemt interacts with p53-binding (1074-1406) and clathrin light chain-binding (1267-1513) domains of CHC tagged with myc-epitope. **b.** Immunoprecipitation of nuclear lysates of NIN3T3 cells with anti-p53 antibodies. The formation of p53 and Pemt complex is confirmed. **c.** Immunoprecipitation of nuclear proteins of liver tissues with anti-p53 antibodies. The complex formation is also confirmed in liver tissues.

Supplemental Figure 7. Down-regulation of clathrin heacy chain (CHC) by overexpression of Pemt. COS-7 cells were transfected with FLAG-Pemt and myc-CHC in different concentrations. **a.** Incremental doses of myc-CHC upregulates the expression of FLAG-Pemt. **b.** The expression of CHC is suppressed by overexpression of FLAG-Pemt in a

dose-dependent manner. **c.** Western blot analysis of liver protein of Pemt+/+, Pemt+/-, Pemt-/- mice under standard (STD) or high fat-high sucrose (HFHS) diet at 25 weeks of age. The expression of CHC increased under HFHS diet and further enhanced by deficiency of Pemt. **d.** Western blot analysis of liver nuclear protein. Liver tissues were excised out at 15 minutes after intraperitoneal injection of 5 units of human insulin and the nuclear proteins were extracted from Pemt+/+ mice under STD or HFHS diet at 25 weeks of age.

Supplemental Figure 8. UCSC genome browser view showing the methylation status of F-box only protein 31 (Fbxo31). Model-based Analysis of ChIP-Seq (MACS)_peak_1995 (Red square) is one of the enrichment regions listed on Supplemental Table 2. At this region, the sequence depth of genomic DNA methylation of Pemt-/- mice (Blue) under high fat-high sucrose diet at 25 weeks of old was significantly increased compared to Pemt+/+ mice (Dark red).

Supplemental Figure 9. UCSC genome browser view showing the methylation status of hepatocyte nuclear factor 4 alpha (HNF4α). Model-based Analysis of ChIP-Seq (MACS)_peak_1221 (Red square) is one of the enrichment regions listed on Supplemental Table 2. At this region, the sequence depth of genomic DNA methylation of Pemt-/- mice (Blue) under high fat-high sucrose diet at 25 weeks of old was significantly increased compared to Pemt+/+ mice (Dark red).

Supplemental Figure 10. Pemt mRNA expression in Japanese patients with non-alcoholic steatohepatitis and simple steatosis. The patients were divided into quartile (Q1, Q2, Q3 and Q4) according to Pemt mRNA levels of liver samples. Lower

quartile of Pemt mRNA (Q2) demonstrated lower body-mass-index (BMI) and platelet counts. All data are means ± SE. n=8 (Q1), 8 (Q2), 10 (Q3), 8 (Q4). *P < 0.05.

Supplemental Figure 11 Uncropped images for the blots shown in Figure 4.

Supplemental Figure 12 Uncropped images for the blots shown in Figures 5 and 6.

Supplemental Figure 13 Uncropped images for the blots shown in Figure S3.

Supplemental Figure 14 Uncropped images for the blots shown in Figure S5.

Supplemental Figure 15 Uncropped images for the blots shown in Figure S5.

Supplemental Figure 16 Uncropped images for the blots shown in Figure S7.

Supplemental Figure 17 Uncropped images for the blots shown in Figure 8.

Supplemental Figure 18 Graphical summary of the results.

Supplemental Figure 1

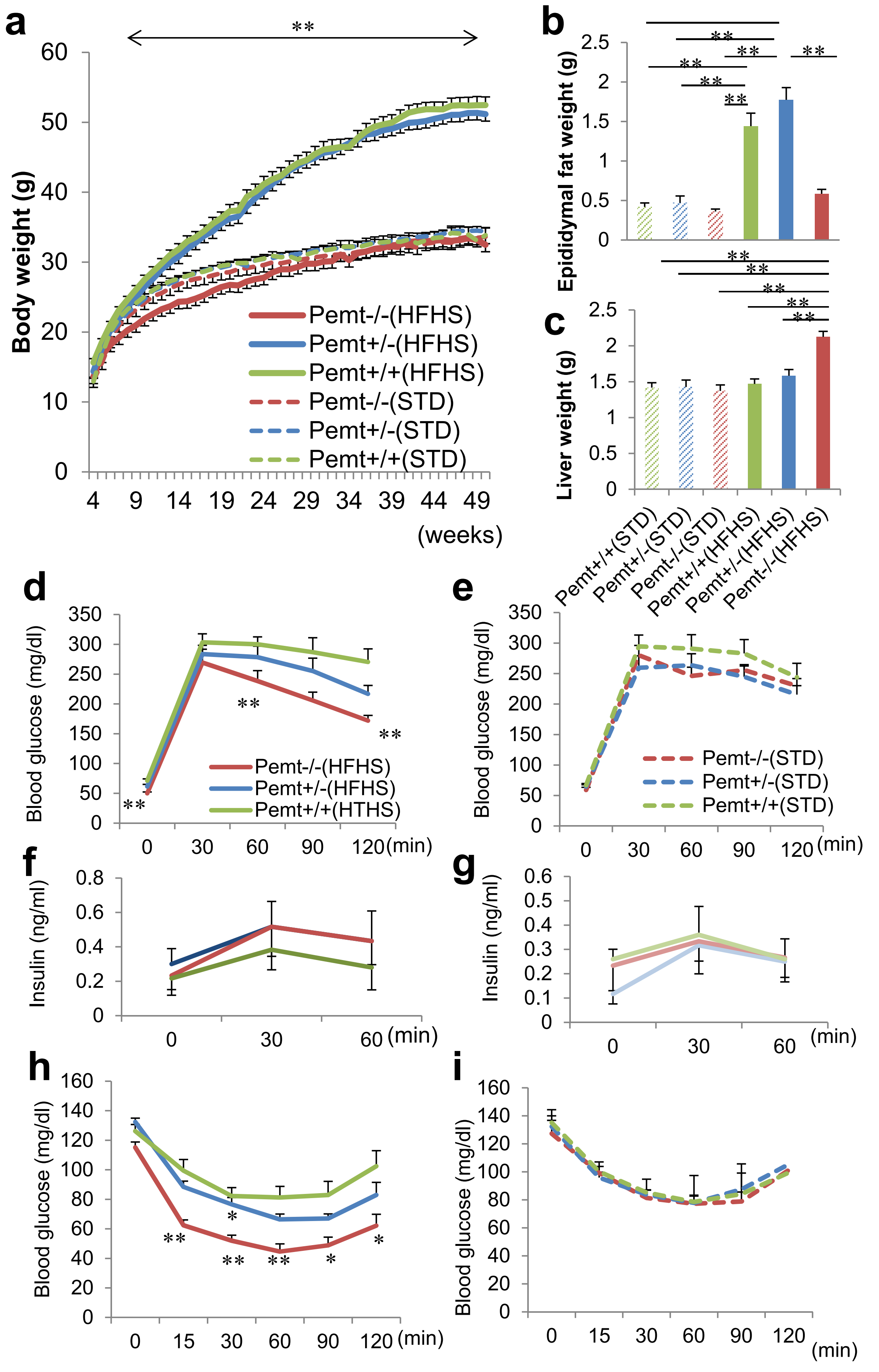

Supplemental Figure 2

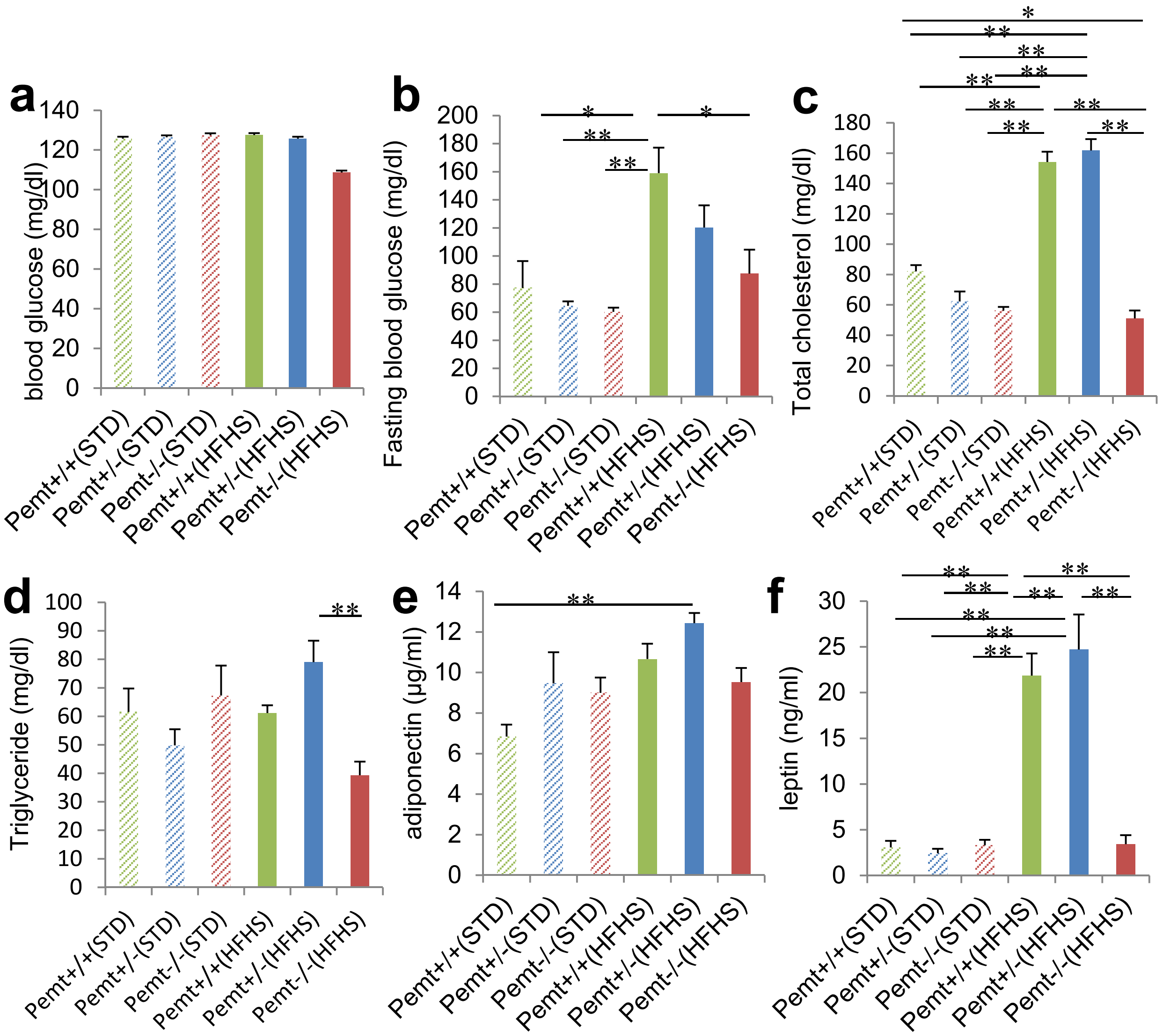

Supplementary Figure 3

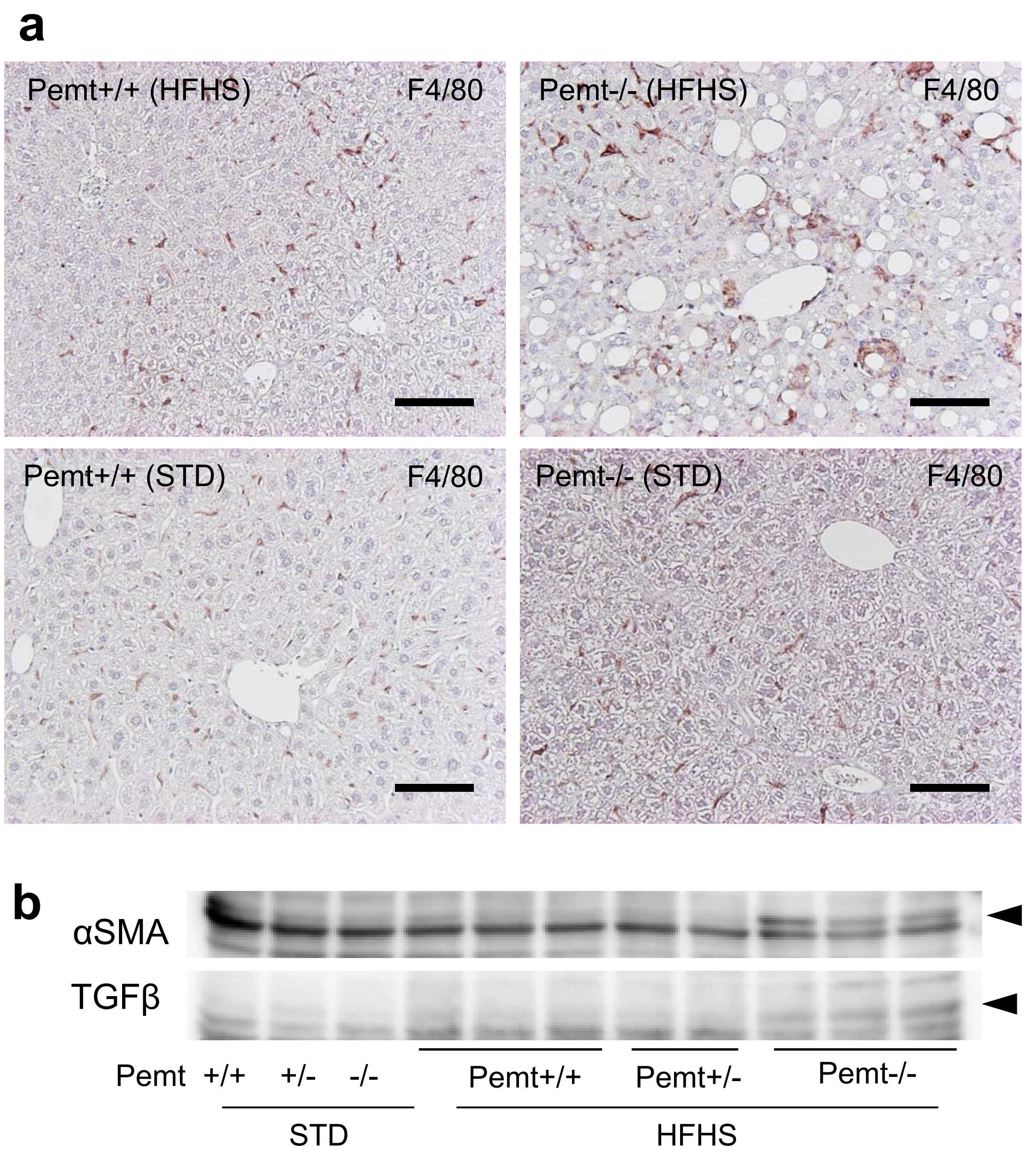

Supplemental Figure 4

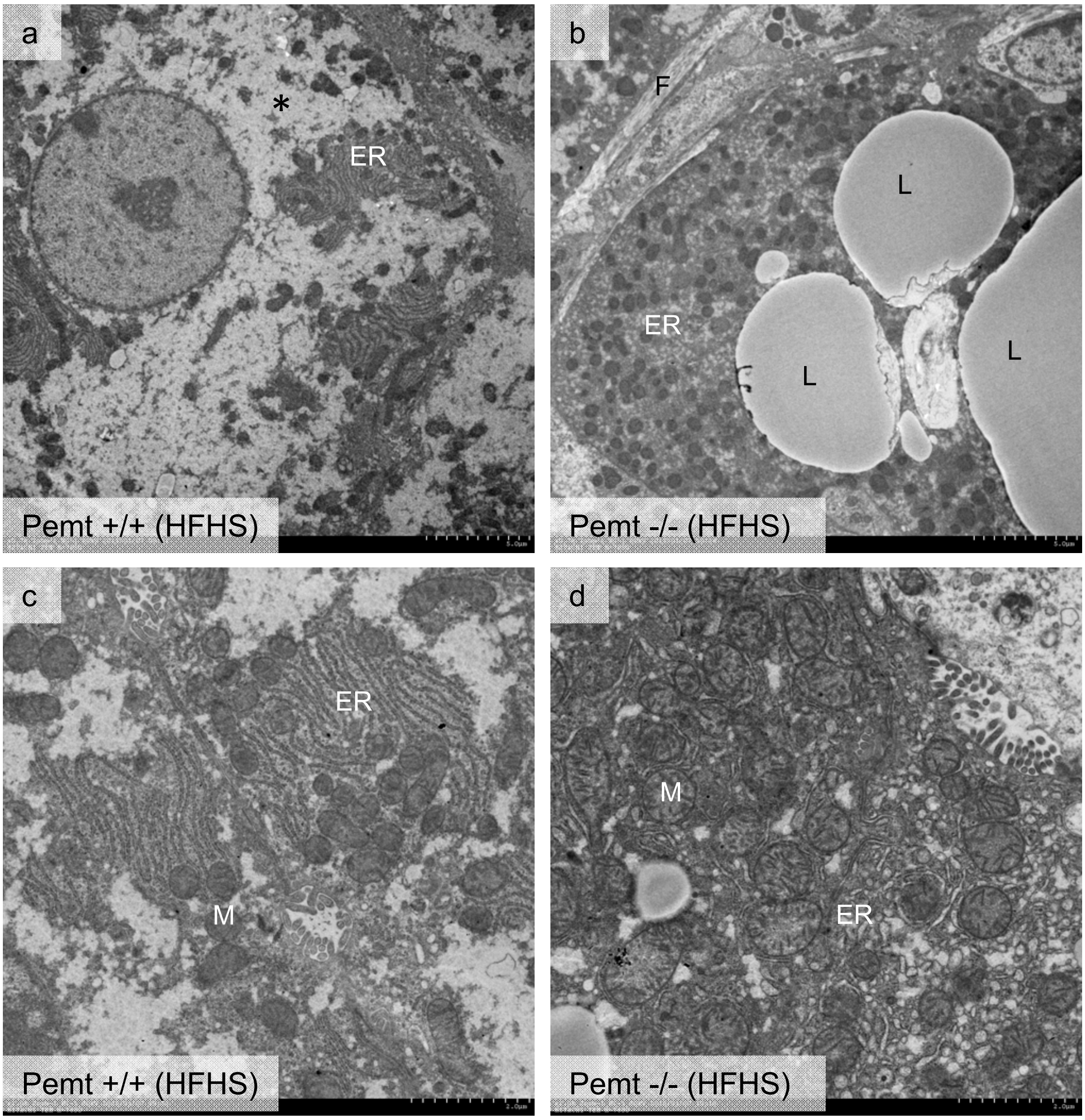

Supplemental Figure 5

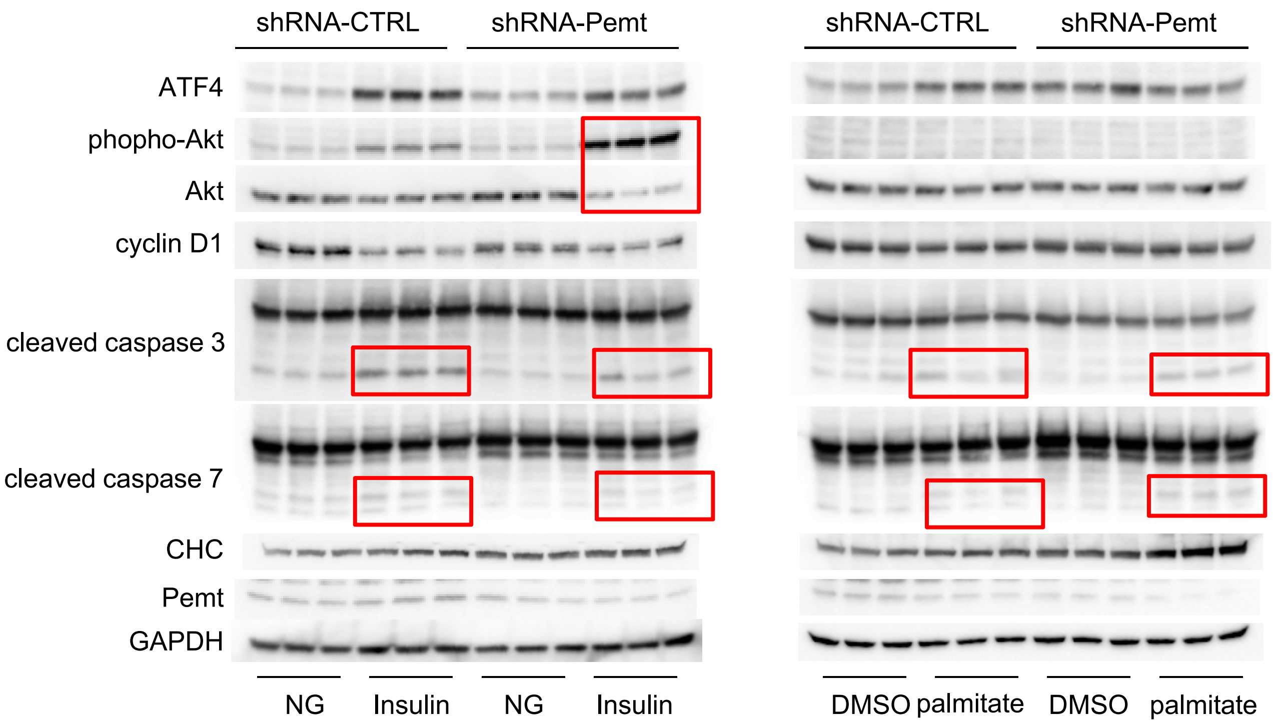

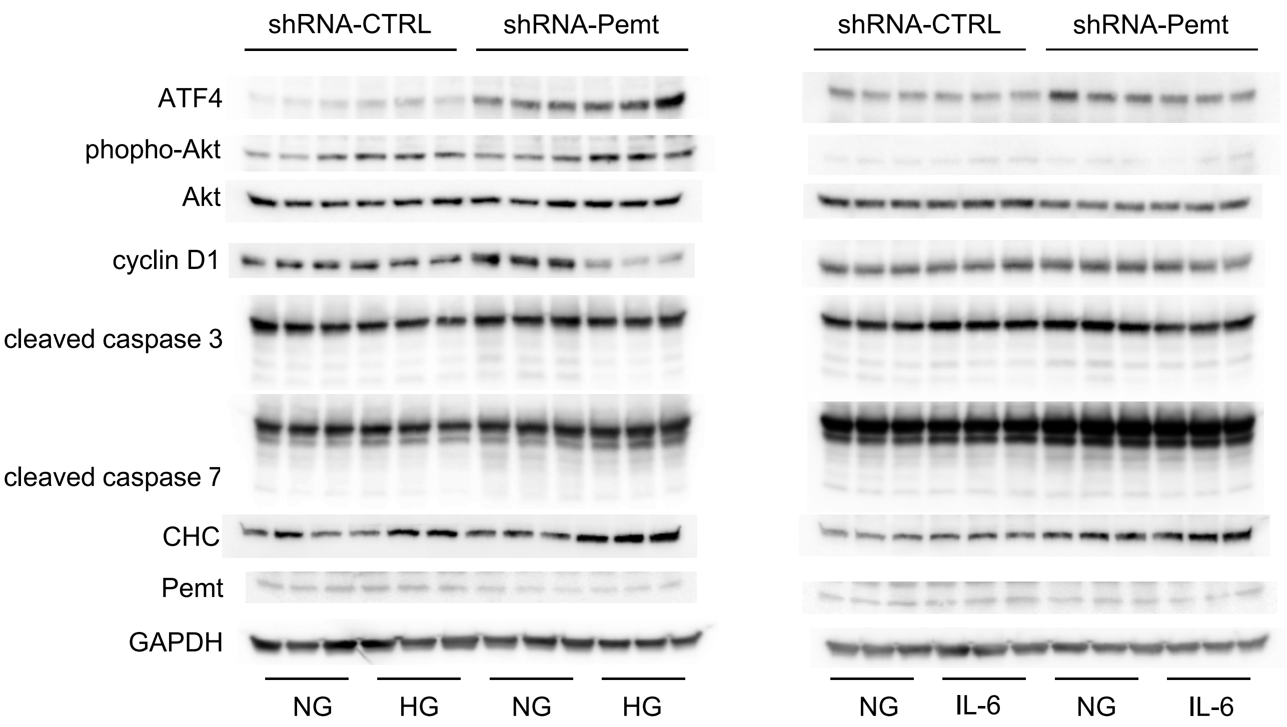

Supplemental Figure 6

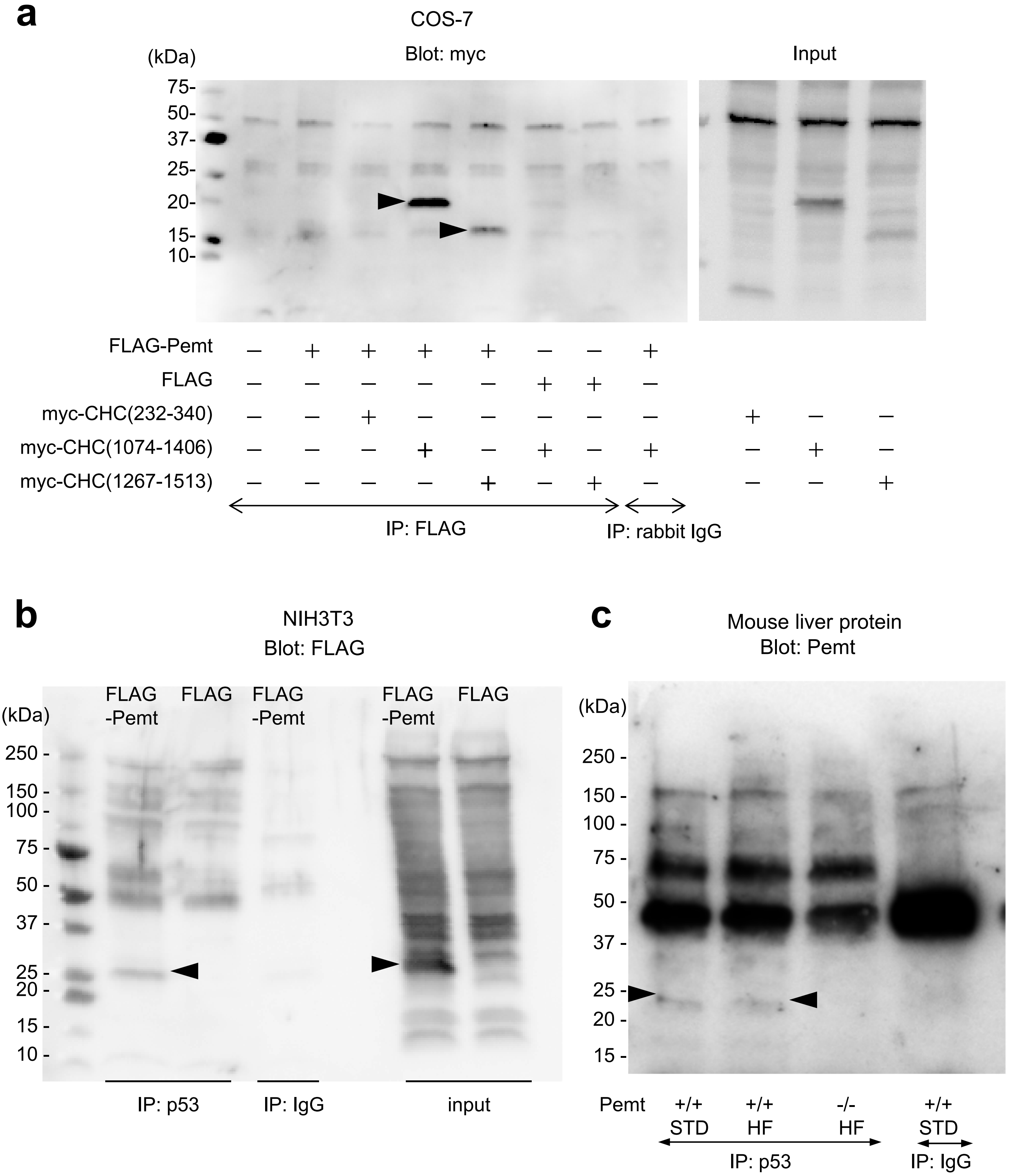

Supplemental Figure 7

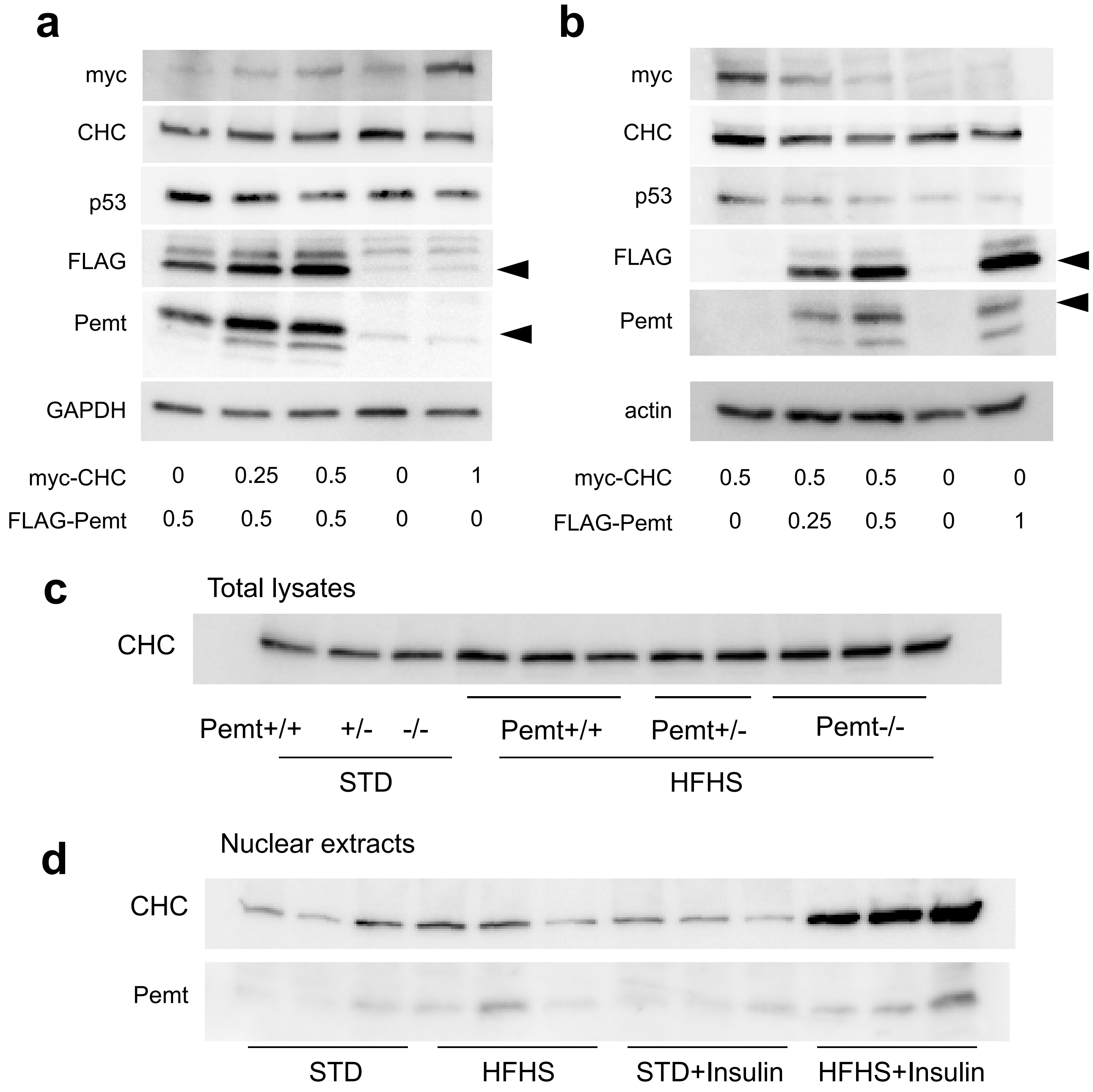

Supplemental Figure 8

Fbxo31

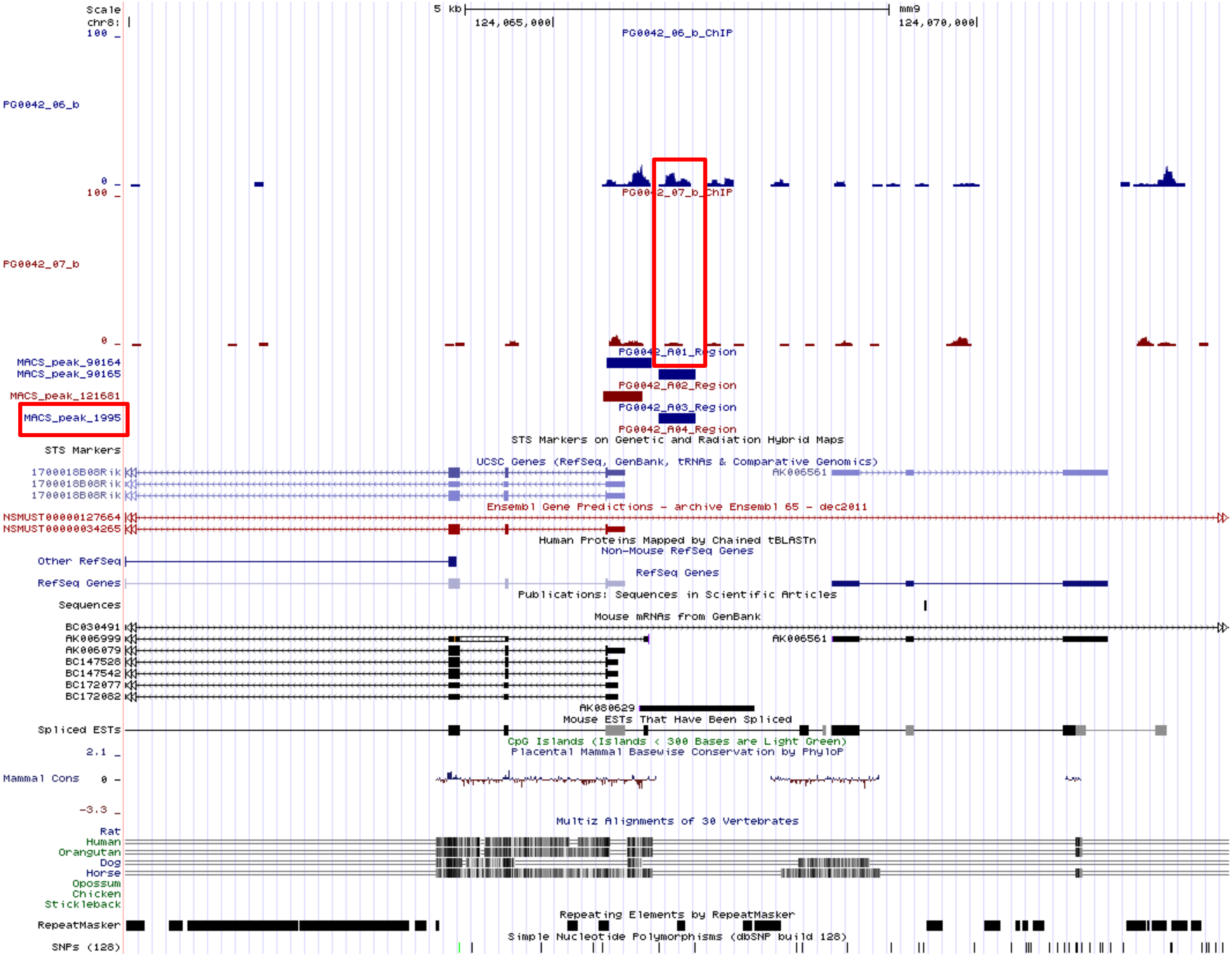

Supplemental Figure 9

HNF4α

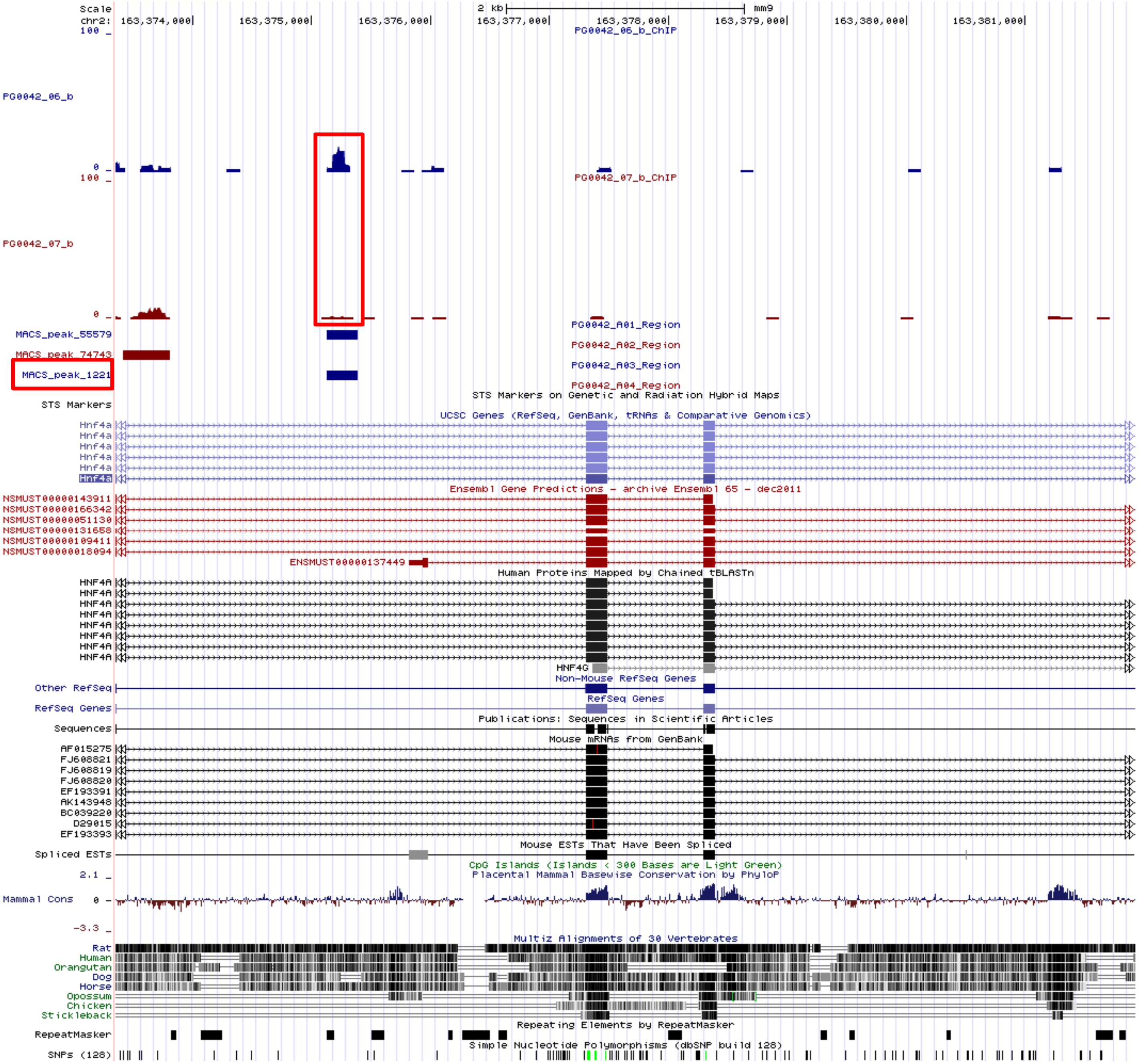

Supplemental Figure 10

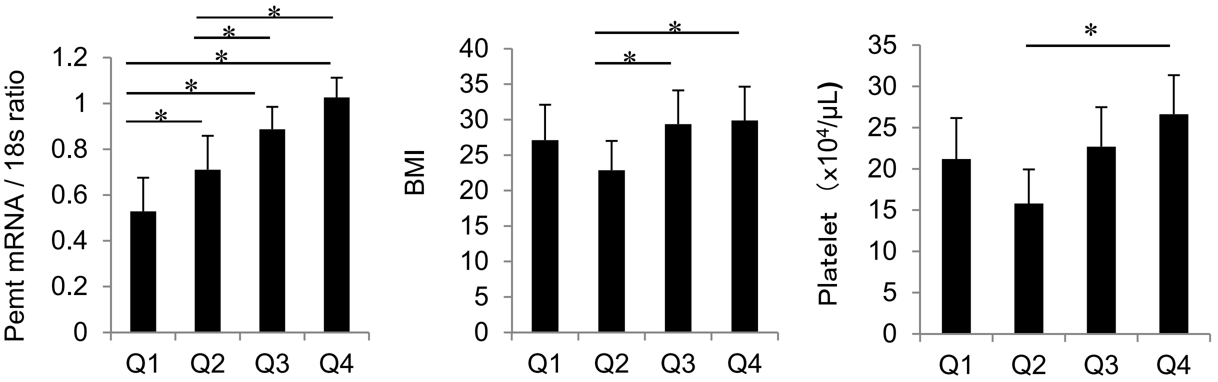

Supplemental Figure 11

Figure 4 Original figure

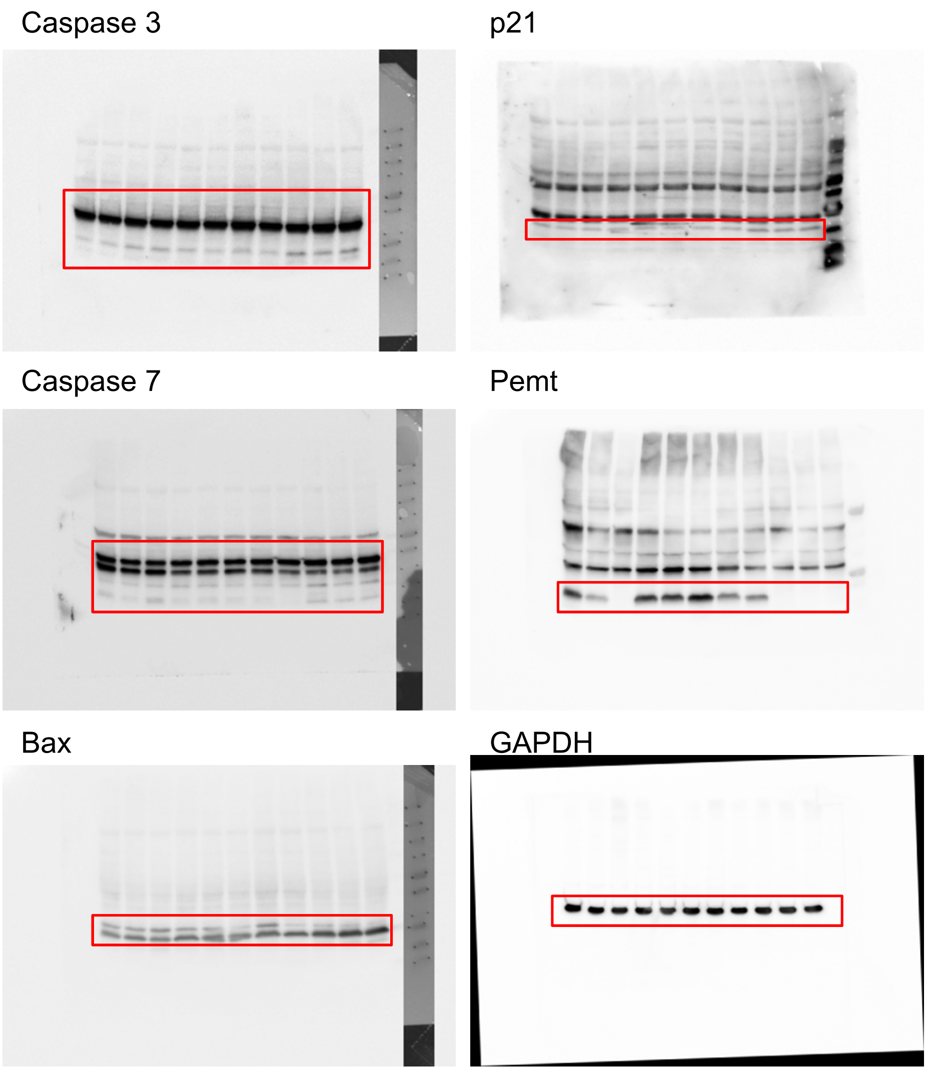

Supplemental Figure 12

Figure 5 Original figure

Cyclin D1 GAPDH

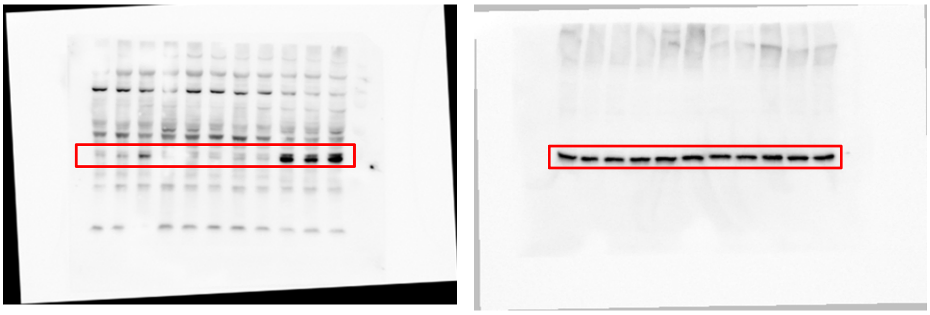

Figure 6 Original figure

a Pemt Histone H3

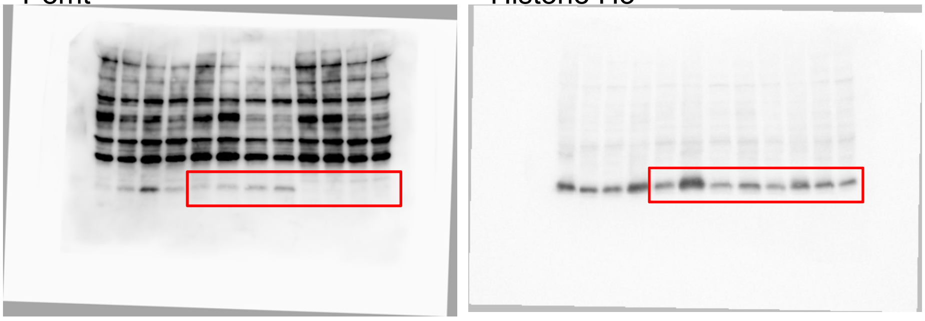

d

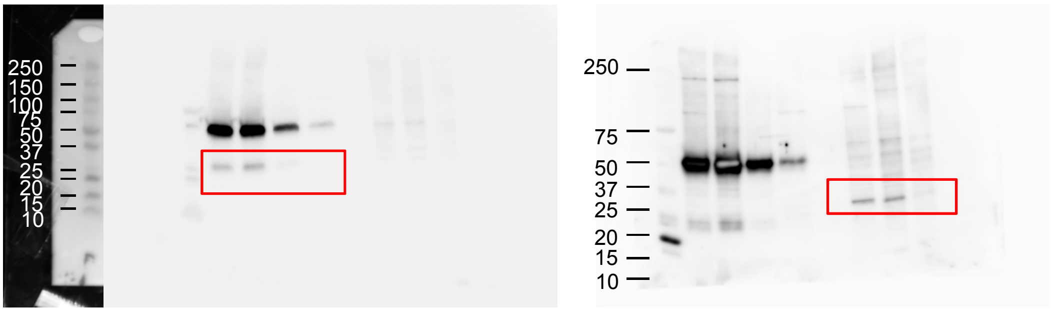

Supplemental Figure 13

Figure S3 Original figure

αSMA

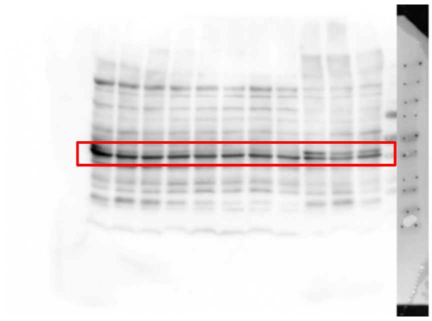

TGFβ

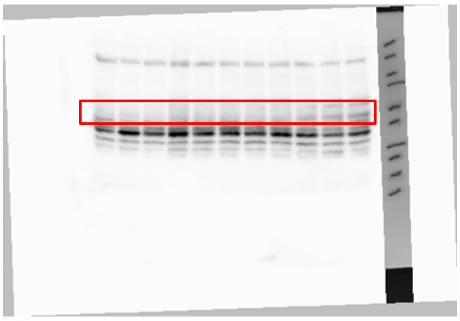

Supplemental Figure 14

Figure S5 Original figure

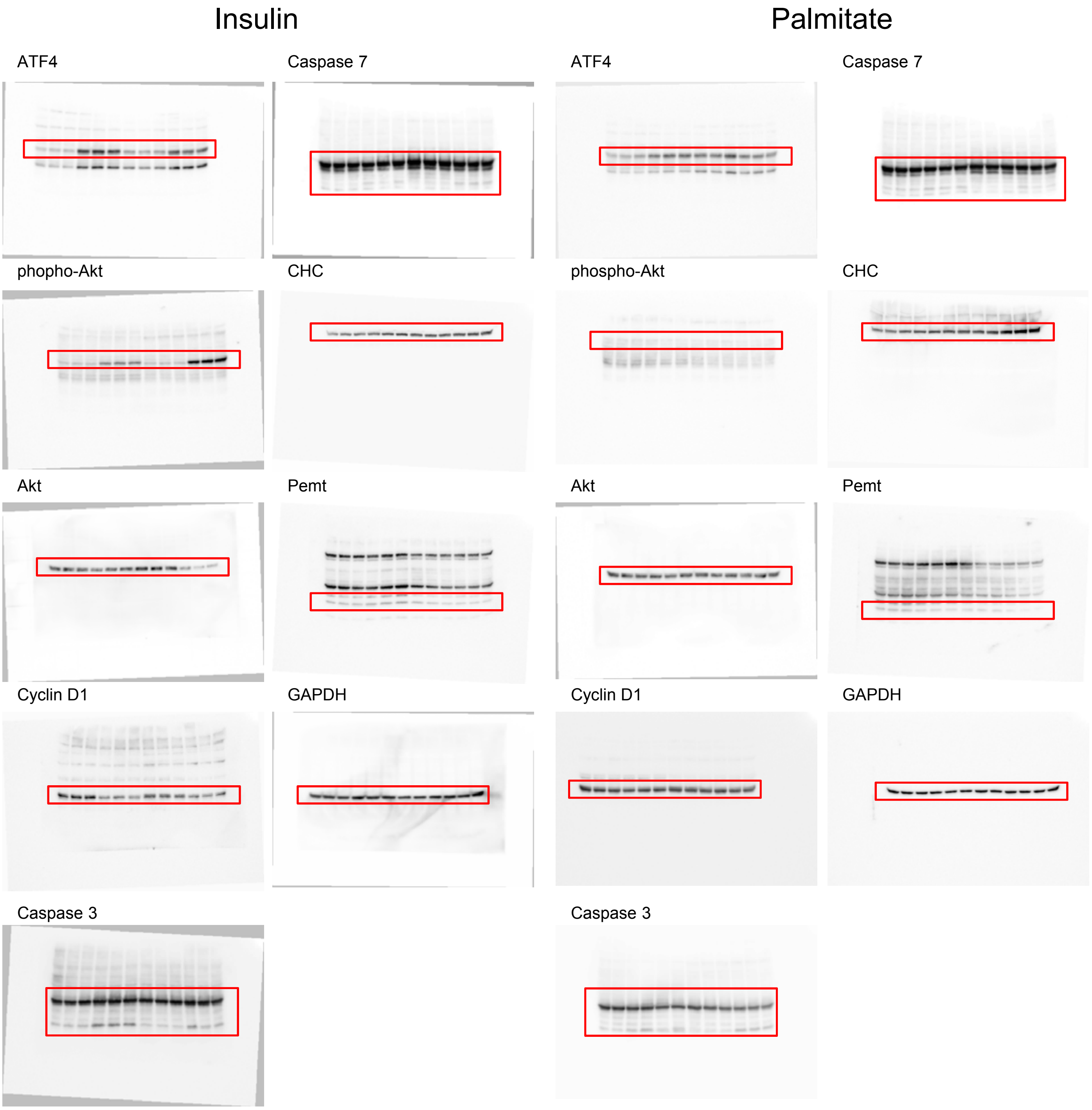

Supplemental Figure 15

Figure S5 Original figure

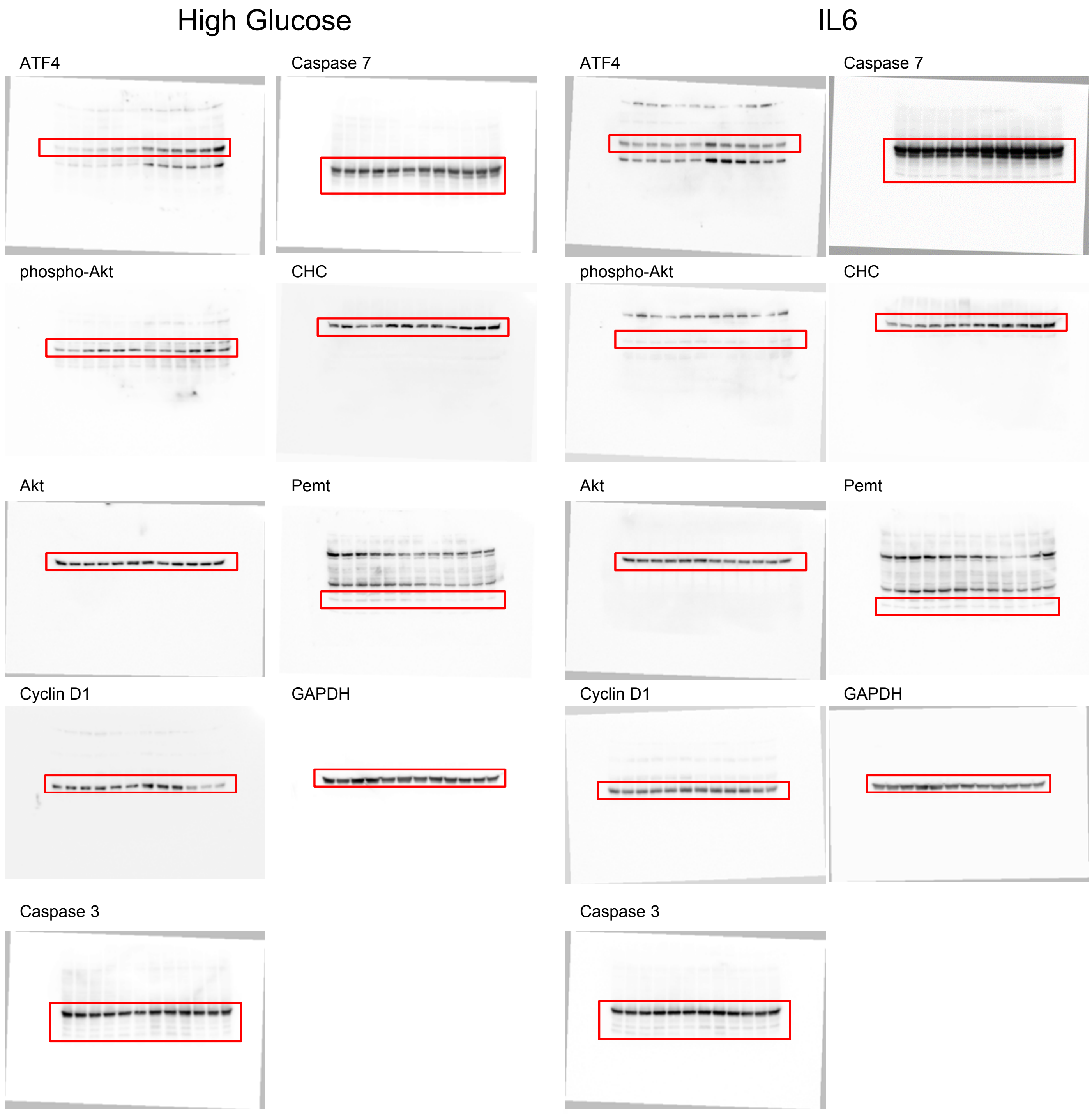

Supplemental Figure 16

Figure S7 Original figure

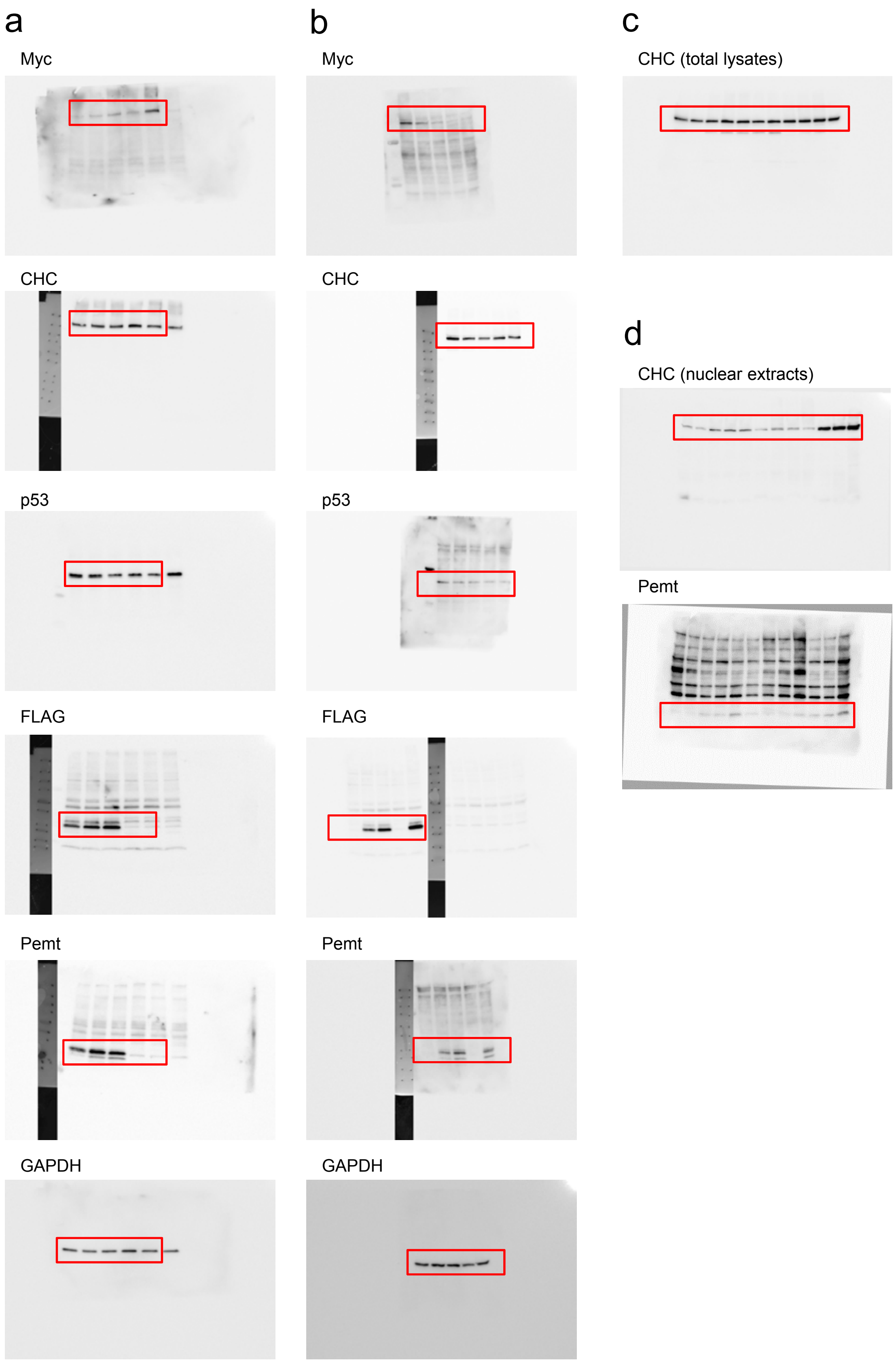

Supplemental Figure 17

Figure 8 Original figure

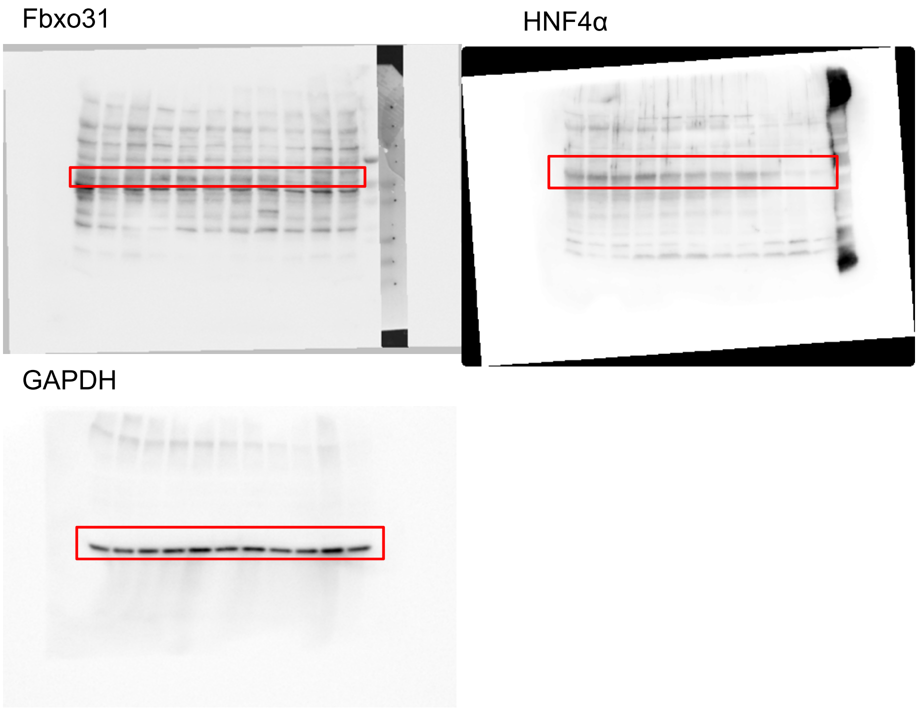

Supplemental Figure 18

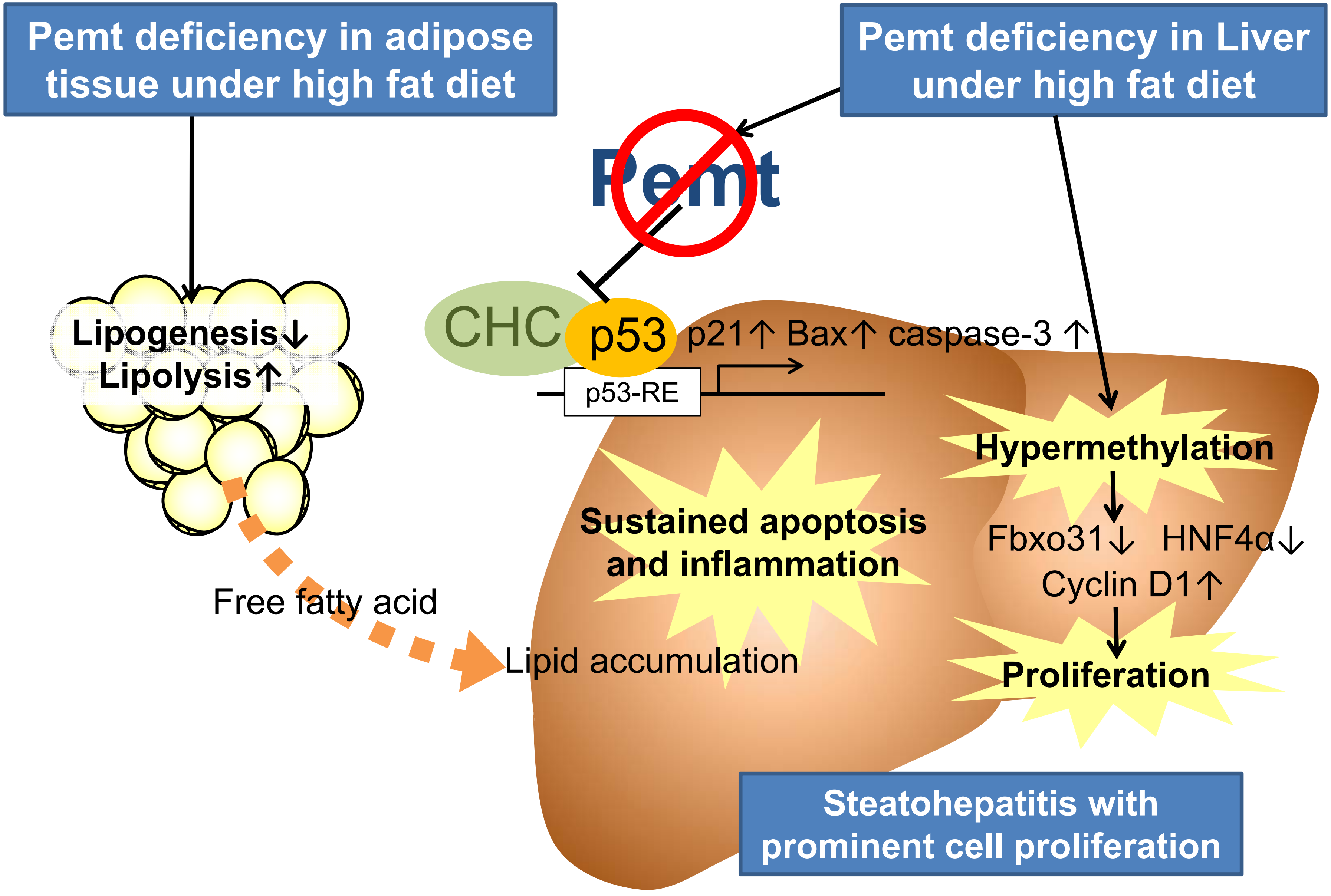

Supplementary Table 1

Primer	
Pemt-*EcoR*I-F	5’-GGGGGGGAATTCAATGAAGAGATCTGGGAACCCGGGA-3’
Pemt-*Xba*I-R	5’-GGGGGGTCTAGATCAGCTCCTCTTGTGGGGCCCGGA-3’
CHC1-*Not*I-F	5’-GGGGGGGCGGCCGCTTGAAGTTGGCACACCACCTACAGGG-3’
CHC1-*Apa*I-R	5’-GGGGGGGGGCCCTTAATTGGTGATGTAAGGAATTATGTT-3’
CHC2-*Not*I-F	5’-GGGGGGGCGGCCGCCCATGGCCCAGATTCTGCCAATTCGT-3’
CHC2-*Apa*I-R	5’-GGGGGGGGGCCCTCACATGCTGTACCCAAAGCCAGG--3’
CHC3-*Not*I-F	5’-GGGGGGGCGGCCGCGGAAATTTGATGTCAATACTTCAGCA-3’
CHC3-*Apa*I-R	5’-GGGGGGGGGCCCTTATCTGTAGTATAGTTCCACATTGGC-3’
CHC4-*Not*I-F	5’-GGGGGGGCGGCCGCTCCGTCTTGCTCAGATGTGTGGACTT-3’
CHC4-*Apa*I-R	5’-GGGGGGGGGCCCTTAATAAGCAGCAATTCTCCTGAACTC--3’
p53-HA-F	5’-GGGGGGGCCACCATGGAGGAGCCGCAGTCAGAT-3’
p53-HA-R	5’-GGGGGGTCAAGCGTAATCTGGAACATCGTATGGGTAGTCTGAGTCAGGCCCTTCTGT-3’
*Hind*III-p53-F	5’-GGGGGGAAGCTTGCCACCATGGAGGAGCCGCAGTCAGAT-3’
HA-*Xho*I-R	5’-GGGGGGCTCGAGTCAAGCGTAATCTGGAACATCGTATGGGTA-3’
FLAG-Pemt-HA-F	5’-GGGGGGGCCACCATGGACTACAAGGACGACGATGACAAAAAGAGATCTGGGAACCCGGGA-3’
FLAG-Pemt-HA-R	5’-GGGGGGTCAAGCGTAATCTGGAACATCGTATGGGTAGCTCCTCTTGTGGGGCCCGGA-3’
*Hind*III-FRAG-F	5’-GGGGGGAAGCTTGCCACCATGGACTACAAGGACGACGATGACAAA-3’
HA-*Xho*1-R	5’-GGGGGGCTCGAGTCAAGCGTAATCTGGAACATCGTATGGGTA-3’

Supplementary Table 2 List of genes with hypermethylated genomic DNA and reduced mRNA expression in Pemt-/- mice compared to Pemt+/+ fed high fat-high sucrose chow.

														mRNA expression ratio (Pemt-/-(HFHS) vs Pemt+/+(HFHS) Log2 ratio					
Region name	Position	Length	Summit	Tags	-10log10(pvalue)	Fold_enrichment	FDR(%)	RefseqID	Status	GeneID	Symbol	Definition	Distance(bp)	1 week	2 week	3 week	4 week	Pearson correlatior	coefficient of determination
MACS_peak_1437	chr4:88335056-88335560	505	128	9	55	16.42	100	NM_010503.2	UP	15965	Ifna2	interferon alpha 2	5373	0.83	0.91	0.41	0.24	-0.23	0.39
MACS_peak_845	chr17:12766866-12767234	369	166	9	56.23	10.84	100	NM_013667.2	UP	20518	Slc22a2	solute carrier family 22 (organic cation transporter), member 2	9820	0.91	0.61	0.1	0.33	-0.23	0.87
MACS_peak_788	chr16:33621596-33621937	342	149	17	83.26	10.6	100	NM_134251.2	INCLUDE	171286	Slc12a8	solute carrier family 12 (potassium/chloride transporters), mem	0	0.91	0.96	0.34	0.49	-0.19	0.82
MACS_peak_112	chr1:176979729-176980084	356	194	9	53.19	11.03	100	NM_011880.3	DOWN	24012	Rgs7	regulator of G protein signaling 7	9122	0.71	0.43	0.45	0.1	-0.18	0.16
MACS_peak_1221	chr2:163375130-163375391	262	131	9	59.3	9.13	100	NM_008261.2	INCLUDE	15378	Hnf4a	hepatic nuclear factor 4, alpha	0	0.97	0.61	0.69	0.42	-0.16	0.75
MACS_peak_249	chr10:127159015-127159368	354	176	10	76.55	15.49	100	NM_009312.2	UP	21334	Tac2	tachykinin 2	3079	0.76	0.19	0.62	0.1	-0.16	0.00
MACS_peak_1206	chr2:155361261-155361697	437	207	8	51.46	12.31	100	NM_019811.3	INCLUDE	60525	Acss2	acyl-CoA synthetase short-chain family member 2	0	0.51	0.59	0.23	0.17	-0.14	0.69
MACS_peak_1995	chr8:124066250-124066683	434	222	11	52.71	8.96	100	NM_133765.3	DOWN	76454	Fbxo31	F-box protein 31	9195	0.79	0.69	0.36	0.44	-0.14	0.79
MACS_peak_1348	chr3:135486084-135486497	414	258	9	62.6	16.42	100	NM_026228.4	UP	67547	Slc39a8	solute carrier family 39 (metal ion transporter), member 8	1957	0.89	0.98	0.73	0.54	-0.13	0.62
MACS_peak_734	chr15:85868509-85868805	297	148	9	78.57	17.43	100	NM_009886.2	UP	12614	Celsr1	cadherin, EGF LAG seven-pass G-type receptor 1 (flamingo ho	4302	0.99	0.67	0.63	0.62	-0.12	0.06
MACS_peak_1879	chr7:150614220-150614703	484	111	9	53.19	11.25	100	NM_008434.2	DOWN	16535	Kcnq1	potassium voltage-gated channel, subfamily Q, member 1	1273	0.43	0.79	0.5	0.24	-0.09	0.77
MACS_peak_1133	chr2:69130200-69130511	312	156	13	56.57	7.85	100	NM_021022.3	INCLUDE	27413	Abcb11	ATP-binding cassette, sub-family B (MDR/TAP), member 11	0	0.77	0.84	0.66	0.56	-0.08	0.24
MACS_peak_190	chr10:70687437-70687775	339	186	8	51.22	11.07	100	NM_009360.4	DOWN	21780	Tfam	transcription factor A, mitochondrial	449	0.98	0.93	0.75	0.81	-0.07	0.75
MACS_peak_1209	chr2:155720125-155720649	525	123	13	57.95	11.07	100	NM_018888.3	INCLUDE	56046	Uqcc	ubiquinol-cytochrome c reductase complex chaperone, CBP3 h	0	0.95	0.84	0.98	0.69	-0.06	0.71
MACS_peak_1976	chr8:106639382-106639891	510	372	13	53.79	11.85	100	NM_009868.4	INCLUDE	12562	Cdh5	cadherin 5	0	0.88	0.53	0.45	0.72	-0.06	0.40
MACS_peak_1894	chr8:13072286-13072668	383	234	18	75.95	10.09	100	NM_025834.3	INCLUDE	66901	Proz	protein Z, vitamin K-dependent plasma glycoprotein	0	0.77	0.81	0.71	0.65	-0.05	0.72
MACS_peak_1538	chr5:49120965-49121544	580	445	14	59.23	12	100	NM_030265.3	INCLUDE	80334	Kcnip4	Kv channel interacting protein 4	0	0.48	0.56	0.48	0.37	-0.04	0.01
MACS_peak_1840	chr7:104561691-104562174	484	299	15	68.01	10.31	100	NM_009381.2	INCLUDE	21835	Thrsp	thyroid hormone responsive SPOT14 homolog (Rattus)	0	0.24	0.23	0.36	0.08	-0.04	0.46
MACS_peak_849	chr17:15607555-15607935	381	213	16	62.14	8.26	100	NM_011185.3	DOWN	19170	Psmb1	proteasome (prosome, macropain) subunit, beta type 1	4749	0.95	0.97	0.83	0.88	-0.04	0.14
MACS_peak_623	chr14:66708189-66708460	272	136	9	89.25	17.43	100	NM_007940.3	INCLUDE	13850	Ephx2	epoxide hydrolase 2, cytoplasmic	0	0.62	0.75	0.56	0.58	-0.03	0.71
MACS_peak_1290	chr3:82806528-82806936	409	152	11	70.81	14.26	100	NM_133862.1	UP	99571	Fgg	fibrinogen gamma chain	4881	0.72	0.83	0.64	0.68	-0.03	0.72
MACS_peak_2045	chr9:53256040-53256403	364	209	20	57.15	5.99	100	NM_007499.2	INCLUDE	11920	Atm	ataxia telangiectasia mutated homolog (human)	0	0.99	0.86	0.94	0.87	-0.03	0.07
MACS_peak_1341	chr3:129561307-129561910	604	460	32	51.26	5.72	100	NM_007686.2	INCLUDE	12630	Cfi	complement component factor i	0	0.71	0.85	0.7	0.67	-0.03	0.25
MACS_peak_1616	chr5:130559279-130559562	284	142	10	62.89	11	100	NM_013837.1	INCLUDE	22021	Tpst1	protein-tyrosine sulfotransferase 1	0	0.81	0.67	0.76	0.7	-0.02	0.02
MACS_peak_542	chr13:94428836-94429076	241	120	8	53.57	7.12	100	NM_022884.2	INCLUDE	64918	Bhmt2	betaine-homocysteine methyltransferase 2	0	0.78	0.86	0.77	0.73	-0.02	0.35
MACS_peak_127	chr1:194304163-194304446	284	142	9	54.64	9.9	100	NM_010600.2	INCLUDE	16510	Kcnh1	potassium voltage-gated channel, subfamily H (eag-related), me	0	0.36	0.08	0.32	0.2	-0.02	0.02
MACS_peak_1812	chr7:70799899-70800347	449	324	8	50.54	12.31	100	NM_130880.1	INCLUDE	170711	Otud7a	OTU domain containing 7A	0	0.65	0.97	0.95	0.58	-0.02	0.03
MACS_peak_687	chr15:27524249-27524604	356	166	10	52.79	9.96	100	NM_020332.4	INCLUDE	11732	Ank	progressive ankylosis	0	0.95	0.68	0.84	0.82	-0.02	0.49
MACS_peak_251	chr10:128367392-128367607	216	108	8	58.32	6.69	100	NM_015740.3	UP	14533	Bloc1s1	biogenesis of lysosome-related organelles complex-1, subunit 1	6812	0.85	0.99	0.89	0.81	-0.02	0.22
MACS_peak_1166	chr2:103294602-103295091	490	237	28	85.3	7.21	100	NM_009804.2	INCLUDE	12359	Cat	catalase	0	0.75	0.75	0.67	0.73	-0.01	0.32
MACS_peak_662	chr15:3350375-3350642	268	134	8	52.3	8.3	100	NM_010284.2	INCLUDE	14600	Ghr	growth hormone receptor	0	0.51	0.9	0.57	0.58	-0.01	0.19
MACS_peak_435	chr12:86616008-86616294	287	143	9	55.07	10	100	NM_025421.2	INCLUDE	66204	Acyp1	acylphosphatase 1, erythrocyte (common) type	0	0.97	0.77	0.9	0.89	-0.01	0.24
MACS_peak_1205	chr2:155011673-155011909	237	118	12	78.87	7.34	100	NM_008395.2	INCLUDE	16396	Itch	itchy, E3 ubiquitin protein ligase	0	0.96	0.88	0.87	0.93	-0.01	0.14
MACS_peak_1180	chr2:118364973-118365318	346	160	9	51.79	10.33	100	NM_138313.3	INCLUDE	171543	Bmf	BCL2 modifying factor	0	0.44	0.06	0.85	0.15	-0.01	0.09
MACS_peak_2037	chr9:45721993-45722285	293	146	8	70.55	16.42	100	NM_008794.2	INCLUDE	18554	Pcsk7	proprotein convertase subtilisin/kexin type 7	0	0.9	0.98	0.98	0.88	-0.01	0.23
MACS_peak_994	chr18:61449422-61449852	431	216	16	62.14	7.34	100	NM_133249.2	DOWN	170826	Ppargc1b	peroxisome proliferative activated receptor, gamma, coactivatoı	7937	0.54	0.55	0.53	0.53	-0.01	0.02
MACS_peak_1158	chr2:94248499-94248890	392	118	9	53.19	10.63	100	NM_007466.2	DOWN	11800	Api5	apoptosis inhibitor 5	2993	0.91	0.94	0.89	0.94	0.00	0.03
MACS_peak_702	chr15:52554640-52554951	312	156	11	60.68	11.51	100	NM_027212.2	INCLUDE	69790	Med30	mediator complex subunit 30	0	0.81	0.93	0.91	0.84	0.01	0.45
MACS_peak_2142	chrX:97960347-97960667	321	160	11	68.45	13.68	100	NM_016747.2	UP	53310	Dlg3	discs, large homolog 3 (Drosophila)	2418	0.62	0.84	0.7	0.72	0.02	0.04
MACS_peak_1612	chr5:125829534-125829852	319	159	9	68.26	17.43	100	NM_016741.1	UP	20778	Scarb1	scavenger receptor class B, member 1	8090	0.76	0.93	0.81	0.86	0.02	0.03
MACS_peak_111	chr1:176819224-176819504	281	140	10	103.32	20.52	100	NM_011825.1	INCLUDE	23893	Grem2	gremlin 2 homolog, cysteine knot superfamily (Xenopus laevis)	0	0.79	0.89	0.72	0.91	0.02	0.09
MACS_peak_1104	chr2:34912635-34913053	419	141	8	52.9	12.31	100	NM_010406.1	INCLUDE	15139	Hc	hemolytic complement	0	0.59	0.7	0.71	0.65	0.02	0.20
MACS_peak_946	chr18:9873595-9873967	373	215	9	53.19	10.11	100	NM_130449.2	INCLUDE	140792	Colec12	collectin sub-family member 12	0	0.78	0.67	0.67	0.85	0.02	0.12
MACS_peak_2006	chr8:129851742-129852135	394	158	10	61.82	12.21	100	NM_00112285	INCLUDE	93742	Pard3	par-3 (partitioning defective 3) homolog (C. elegans)	0	0.76	0.49	0.15	0.95	0.02	0.10
MACS_peak_1979	chr8:108094784-108095078	295	147	9	68.93	13.94	100	NM_007427.2	UP	11604	Agrp	agouti related protein	2586	0.57	0.33	0.99	0.43	0.02	0.01
MACS_peak_1311	chr3:95038532-95038896	365	142	11	52.71	8.48	100	NM_134253.1	DOWN	171388	Bnipl	BCL2/adenovirus E1B 19kD interacting protein like	6318	0.6	0.4	0.88	0.52	0.02	0.08
MACS_peak_908	chr17:72738020-72738230	211	105	9	112.99	14.71	100	NM_007439.2	INCLUDE	11682	Alk	anaplastic lymphoma kinase	0	0.49	0.69	0.48	0.66	0.03	0.05
MACS_peak_1605	chr5:121593389-121593682	294	147	14	54.15	6.38	100	NM_00110999	INCLUDE	19247	Ptpn11	protein tyrosine phosphatase, non-receptor type 11	0	0.91	0.42	0.79	0.89	0.03	0.14
MACS_peak_1349	chr3:137881274-137881765	492	346	11	52.71	10.16	100	NM_009626.4	INCLUDE	11529	Adh7	alcohol dehydrogenase 7 (class IV), mu or sigma polypeptide	0	0.68	0.87	0.98	0.76	0.04	0.42
MACS_peak_1968	chr8:93984684-93985018	335	167	8	60.16	16.31	100	NM_011936.2	INCLUDE	26383	Fto	fat mass and obesity associated	0	0.8	0.97	0.9	0.95	0.04	0.59
MACS_peak_1736	chr6:119676730-119677237	508	160	11	59.99	12.39	100	NM_053204.2	INCLUDE	111173	Erc1	ELKS/RAB6-interacting/CAST family member 1	0	0.63	0.87	0.96	0.74	0.04	0.58
MACS_peak_717	chr15:75723765-75724103	339	121	8	60.29	14.37	100	NM_023240.2	DOWN	66656	Eef1d	eukaryotic translation elongation factor 1 delta (guanine nucleo	1126	0.81	0.99	0.96	0.99	0.05	0.01
MACS_peak_1299	chr3:87982310-87982778	469	321	17	68.93	10.9	100	NM_133665.3	DOWN	17261	Mef2d	myocyte enhancer factor 2D	9221	0.58	0.64	0.59	0.77	0.05	0.47
MACS_peak_1220	chr2:163208420-163208736	317	158	13	50.7	7.98	100	NM_021566.1	INCLUDE	59091	Jph2	junctophilin 2	0	0.41	0.5	0.08	0.74	0.06	0.59
MACS_peak_1924	chr8:47923961-47924209	249	124	9	100.07	17.36	100	NM_008391.4	INCLUDE	16363	Irf2	interferon regulatory factor 2	0	0.68	0.64	0.88	0.83	0.07	0.12
MACS_peak_1687	chr6:58582770-58583365	596	208	15	60.77	12.7	100	NM_011920.3	INCLUDE	26357	Abcg2	ATP-binding cassette, sub-family G (WHITE), member 2	0	0.72	0.58	0.72	0.92	0.07	0.55
MACS_peak_264	chr11:11618371-11618880	510	158	12	80.15	21.73	100	NM_00102559	INCLUDE	22778	Ikzf1	IKAROS family zinc finger 1	0	0.27	0.92	0.68	0.65	0.09	0.07
MACS_peak_1689	chr6:64013625-64014102	478	325	12	60.31	11.11	100	NM_008167.2	INCLUDE	14804	Grid2	glutamate receptor, ionotropic, delta 2	0	0.62	0.62	0.6	0.97	0.10	0.84
MACS_peak_160	chr10:41906655-41907197	543	406	17	56.45	6.86	100	NM_019740.2	INCLUDE	56484	Foxo3	forkhead box O3	0	0.37	0.43	0.5	0.82	0.14	0.19
MACS_peak_281	chr11:35958621-35958974	354	192	10	59.31	11.62	100	NM_011856.3	INCLUDE	23964	Odz2	odd Oz/ten-m homolog 2 (Drosophila)	0	0.22	0.82	0.55	0.88	0.17	0.54

Tags	The number of read sequences that align to a unique range of enrichment region
FDR (%)	False Dicovery Rate
RedSeqID	Reference sequence ID founded within 10 kbp from enrichement region
Status	Positinal relationship between RefSeqID and enrichment region
Distance	Lengh between RefSeq and enchment region

Supplementary Table 3

PEMT mRNA expression in livers and clinical features in SS and NASH patients

	SS (n=9)	NASH (n=25)	*P* value
Age (years)	44.4 ± 11.3	50.2 ± 15.1	n.s.
M:F	5 : 4	11 : 14	n.s.
BMI (kg/m^2)	26.8 ± 6.1	27.7 ± 5.0	n.s.
Platelet (x10^4/μL)	23.6 ± 6.4	20.9 ± 8.9	n.s.
AST (IU/L)	42.1 ± 17.8	76.9 ± 51.5	0.058
ALT (IU/L)	69.3 ± 40.9	99.9 ± 90.1	n.s.
γGTP (IU/L)	112.9 ± 93.2	93.3 ± 77.4	n.s.
Total cholesterol (mg/dl)	194.8 ± 56.1	191.2 ± 54.0	n.s.
LDL-cholesterol (mg/dl)	117.8 ± 57.1	117.0 ± 48.3	n.s.
HDL-cholesterol (mg/dl)	56.1 ± 12.8	51.5 ± 15.7	n.s.
Triglyceride (mg/dl)	188.2 ± 83.2	164.8 ± 85.3	n.s.
HbA1c (%)	5.5 ± 0.4	5.6 ± 0.9	n.s.
Fasting plasma glucose (mg/dl)	102.7 ± 8.0	109.7 ± 20.8	n.s.
Immunoreactive insulin	10.0 ± 7.1	18.6 ± 10.7	0.014
HOMA-IR	2.6 ± 1.9	5.5 ± 4.3	0.013
Ferritin (ng/dl)	338.7 ± 362.7	325.6 ± 356.8	n.s.
Liver biopsy samples			
PEMT mRNA / 18s ratio	0.95 ± 0.14	0.74 ± 0.22	0.004

SS: simple steatosis, NASH; non-alcoholic steatohepatitis, BMI; body mass index, HOMA-IR; homeostasis model assessment-Insulin resistance

Supplementary Table 4

PEMT mRNA expression in livers and clinical features on SS and NASH patients

	SS (n=9)	NASH (n=25)	*P* value
Histological factors			
Grade			0.006
1	9	10	
2	0	11	
3	0	5	
4	0	0	
Stage			0.001
1	9	6	
2	0	5	
3	0	10	
4	0	5	
Matteoni classification			0.000
1	6	0	
2	3	0	
3	0	0	
4	0	26	

Supplementary Table 5

Pearson Correlation Coefficient of *PEMT* mRNA expression in liver with clinical features in SS and NASH patients

	correlation coefficient	*P* value
Age (years)	-0.245	0.157
BMI (kg/m^2)	0.247	0.153
Platelet (x10^4/μL)	0.357	0.035
AST (IU/L)	0.095	0.586
ALT (IU/L)	0.301	0.079
γGTP (IU/L)	0.294	0.086
Total cholesterol (mg/dl)	-0.091	0.602
LDL-cholesterol (mg/dl)	-0.082	0.649
HDL-cholesterol (mg/dl)	-0.166	0.340
Triglyceride (mg/dl)	0.298	0.082
HbA1c (%)	0.138	0.431
Fasting plasma glucose (mg/dl)	-0.048	0.784
Immunoreactive insulin	-0.138	0.428
HOMA-IR	-0.183	0.300
Ferritin (ng/dl)	-0.025	0.892
